# Whole exome sequencing reveals an *FCGBP* variant associated with spontaneous intraabdominal hemorrhage in severe acute pancreatitis

**DOI:** 10.1002/imo2.52

**Published:** 2025-01-09

**Authors:** Qiu‐Yi Tang, Yue‐Peng Hu, Qi Yang, Jing Zhou, Jing‐Zhu Zhang, Jie Yang, Hai‐Bin Hao, Gang Li, Bai‐Qiang Li, Lu Ke, Zhi‐Hui Tong, Yu‐Xiu Liu, Evan Yi‐Wen Yu, Wei‐Qin Li

**Affiliations:** ^1^ School of Medicine Southeast University Nanjing China; ^2^ Department of Critical Care Medicine, Jinling Hospital, Affiliated Hospital of Medical School Nanjing University Nanjing China; ^3^ Department of General Surgery Xinhua Hospital Affiliated to Shanghai Jiao Tong University School of Medicine Shanghai China; ^4^ Department of Emergency Medicine The First Affiliated Hospital of Zhengzhou University Zhengzhou China; ^5^ Key Laboratory of Environmental Medicine and Engineering of Ministry of Education, and Department of Epidemiology & Biostatistics, School of Public Health Southeast University Nanjing China; ^6^ Department of Epidemiology, CAPHRI Care and Public Health Research Institute, School of Nutrition and Translational Research in Metabolism Maastricht University Maastricht the Netherlands

**Keywords:** acute pancreatitis, gene‐prognosis association, spontaneous intraabdominal hemorrhage, whole exome sequencing

## Abstract

This study investigated the genetic basis of spontaneous intraabdominal hemorrhage (SIH) in severe acute pancreatitis (SAP) to facilitate the development of more effective treatments for this life‐threatening complication. A four‐phase study was conducted with a large cohort of acute pancreatitis (AP) patients (*n* = 600). In the first phase, whole‐exome sequencing identified a specific exonic variant (rs1326680184) in the human Fc gamma binding protein (*FCGBP*) gene consistently associated with SIH. The second phase employed serum ELISA tests, revealing that this variant altered FCGBP protein levels, increasing susceptibility to SIH. In the third phase, functional validation was performed through: (i) in vivo experiments using a *Fcgbp*‐knockdown mouse model demonstrated that reduced *Fcgbp* expression exacerbated AP severity and increased the risk of hemorrhage; and (ii) in vitro experiments with *FCGBP*‐knockdown in human vascular fibroblasts showed that decreased *FCGBP* expression destabilized the vascular wall, leading to vascular injury in SAP. Finally, the fourth phase compared clinical characteristics of *FCGBP* rs1326680184 carriers and non‐carriers, finding that carriers exhibited higher risks of severe complications, worse AP prognosis, and demonstrated enhanced diagnostic utility as a predictive indicator. These findings provide critical insights into the genetic basis of SIH in SAP, paving the way for precision therapies and effective prognostic tools to improve AP management and early intervention.

## Highlights

- A large cohort of acute pancreatitis patients (*n* = 600) underwent whole‐exome sequencing.
- Genetic mutation in *FCGBP* presents a strong association with the predisposition of spontaneous intraabdominal hemorrhage.
- The incorporation of *FCGBP* mutation as an indicator enhances the early assessment for complications and mortality of acute pancreatitis.

## Graphical abstract

A large cohort of acute pancreatitis patients (*n* = 600) underwent whole‐exome sequencing, which identified a genetic mutation in *FCGBP* strongly associated with a predisposition of spontaneous intraabdominal hemorrhage. Incorporating the *FCGBP* mutation as a clinical indicator enhances the assessment for complications and mortality risk in acute pancreatitis, even in the early phase of the disease.

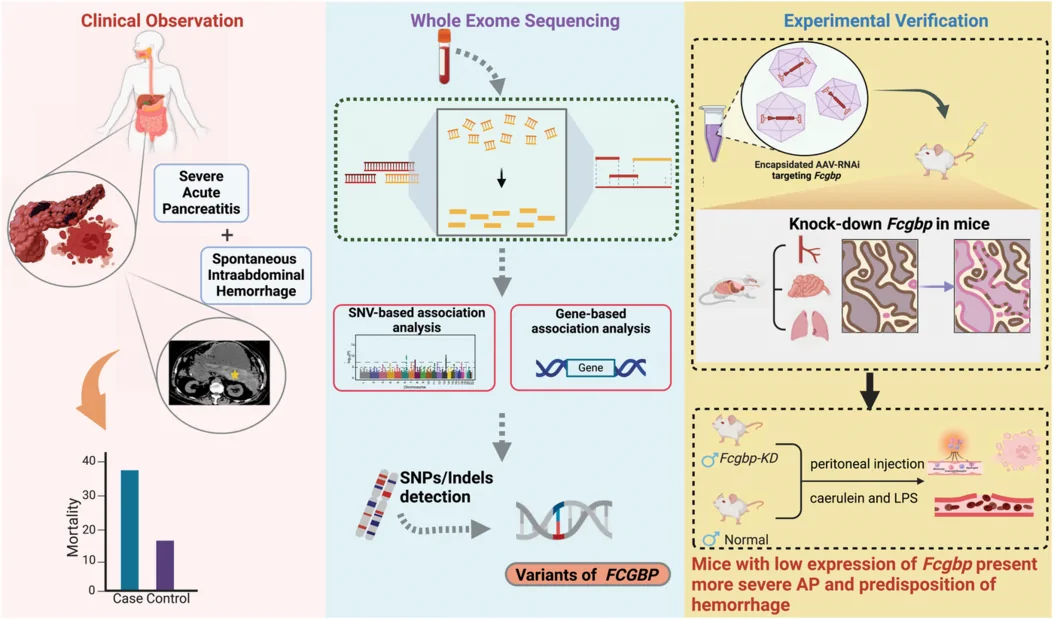

## 1 INTRODUCTION

Acute pancreatitis (AP) is an acute inflammatory condition of the pancreas and a leading cause of acute abdominal diseases worldwide. The annual incidence of AP ranges from 13 to 45 cases per 100,000 individuals, with approximately 20% of cases progressing to severe outcomes, including death [1, 2]. Population‐based cohort studies report an AP‐related mortality rate of 1.16 per 100,000 individuals annually [3]. However, the severe form of AP, often complicated by conditions such as infected pancreatic necrosis (IPN) and acute respiratory distress syndrome (ARDS), is associated with a high mortality rate of over 30.0% [4]. Despite its clinical burden, there are currently no therapeutic agents capable of altering the course of AP, and the mechanisms of its complications remain largely unknown.

As one of the life‐threatening complications, intraabdominal hemorrhage occurs during the course of AP, particularly in severe acute pancreatitis (SAP). This condition significantly increases the risk of abdominal infection, multiple organ dysfunction, and fatal consequences. Several contributing factors, including progressive inflammation, portal hypertension, necrosis, and surgical necrosectomy, have been implicated in intraabdominal hemorrhage [5]. However, the evidence remains inconsistent and controversial.

The causes and pathogenesis of intraabdominal hemorrhage, particularly spontaneous intraabdominal hemorrhage (SIH), have not been well understood. SIH is a critical form of intraabdominal hemorrhage that develops during AP development without prior surgical intervention, presenting unique challenges for early recognition and management. According to Chen et al., AP patients complicated by SIH have worse prognosis, with mortality rates as high as 54.2%, compared to 20.8% in those without SIH [6], identifying high‐risk AP patients before this condition manifests is therefore crucial. Despite its severity, research on SIH in AP patients remains limited, and the risk factors and underlying mechanisms are largely unknown. Genetic variants have been implicated in the development of several AP‐related complications [7, 8, 9, 10, 11], suggesting that genetic predisposition may play a crucial role in the pathogenesis of SIH.

Over the past decade, next‐generation sequencing techniques, particularly whole exome sequencing (WES), have been increasingly used to investigate genetic causes in complex diseases, especially for agnogenic phenotypes [12]. WES offers a powerful tool for uncovering unexplained disorders, enabling the identification of potentially actionable variants and maximizing both clinical and biological exploration. This approach can guide clinical decision‐making and provide valuable insights for follow‐up.

Here, we present the largest WES study to date in AP, investigating the disease‐specific influence of exonic variants on SIH. By unveiling potential causes and mechanisms, we seek to understand the basis of SIH and facilitate the development of more effective strategies for AP prognosis and management.

## 2 RESULTS

### Demographic and clinical characteristics of included participants

**FIGURE 1:**
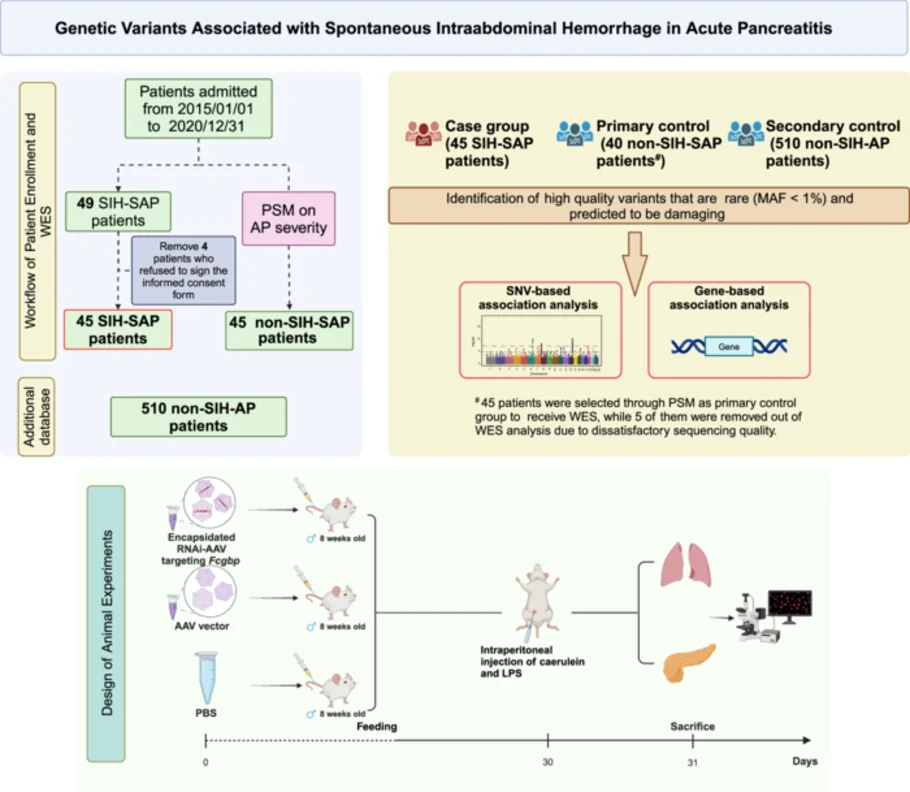
Schematic representation of participant enrolment, bioinformatics analysis, and animal experiments. The study included 45 SIH‐SAP cases, 45 primary non‐SIH‐SAP controls, and 510 secondary non‐SIH‐AP controls. A series of analysis were performed to identify the exome variants related to SIH development. Animal experiments were used to validate the effects of the identified gene in inducing SIH in SAP model. AP, acute pancreatitis patients; LPS, lipopolysaccharides; MAF, minor allele frequency; RNAi‐AAV, adeno‐associated virus packaged with small hairpin RNA; PBS, phosphate buffer saline; SIH, spontaneous intraabdominal hemorrhage; SAP, severe acute pancreatitis.

A detailed description of the study design, workflow, and data processing is illustrated in Figure 1. Among patients admitted with AP to a tertiary medical center (Jinling Hospital, Nanjing, China), 49 patients were determined as having severe or critical complications of AP and diagnosed with SIH (SIH‐SAP). No pedigree relatedness was observed according to the lineage data gained from all included participants.

The primary control (non‐SIH‐SAP) group consisted of 45 SAP patients without SIH, selected using a 1:1 ratio propensity score matching (PSM) based on AP severity. Severity was quantified using the Acute Physiology and Chronic Health Evaluation Ⅱ (APACHE Ⅱ) score and defined as mild, moderate, severe, or critical according to determinants‐based classification (DBC) guideline [13]. As shown in Table 1, there is no difference between the SIH‐SAP and non‐SIH‐SAP groups in terms of median APACHE Ⅱ scores (15.00 vs. 15.00), incidence of acute kidney injury (80.0% vs. 68.8%) and ARDS (82.2% vs. 80.0%).

**TABLE 1:** Demographical and clinical characteristics of participants^a^.

	Overall (*n* = 90)	SIH (*n* = 45)	Control (*n* = 45)	*p*
Age^b^ (median (IQR))	41.00 (31.00, 49.75)	46.00 (33.00, 56.00)	38.00 (30.00, 44.00)	0.007
APACHE Ⅱ (median (IQR))	15.00 (10.00, 18.75)	15.00 (10.00, 18.00)	15.00 (10.00, 19.00)	0.872
BMI (median (IQR))	25.70 (23.00, 28.41)	26.00 (23.44, 28.56)	24.95 (22.40, 28.25)	0.285
Gender (%)				
Male	61 (67.8)	37 (82.2)	24 (53.3)	0.006
Female	29 (32.2)	8 (17.8)	21 (46.7)	
Death (%)	26 (28.9)	16 (35.6)	10 (22.2)	0.245
Etiology of AP (%)				
Hypertriglyceridemia	66 (73.3)	21 (46.7)	45 (100.0)	<0.001
Biliary	20 (22.2)	20 (44.4)	0 (0.0)	
Others	4 (4.4)	4 (8.9)	0 (0.0)	
ARDS (%)	73 (81.1)	37 (82.2)	36 (80.0)	0.999
AKI (%)				
Level 3	45 (50.0)	21 (46.7)	24 (53.3)	0.181
Level 2	4 (4.4)	2 (4.4)	2 (4.4)	
Level 1	18 (20.0)	13 (28.9)	5 (11.1)	
Sepsis (%)	21 (23.3)	15 (33.3)	6 (13.3)	0.045
Hypertension (%)	16 (17.8)	12 (26.7)	4 (8.9)	0.051
Diabetes (%)	18 (20.0)	9 (20.0)	9 (20.0)	0.999
Hyperlipidemia (%)	52 (57.8)	19 (42.2)	33 (73.3)	0.005
PT (median (IQR); second)	14.45 (13.40, 15.70)	14.70 (13.60, 15.70)	14.30 (13.00, 15.70)	0.397
APTT (median (IQR); second)	34.25 (28.83, 43.95)	34.40 (28.50, 47.30)	34.00 (29.60, 41.30)	0.643
INR (median (IQR))	1.26 (1.16, 1.37)	1.27 (1.17, 1.37)	1.23 (1.13, 1.36)	0.375
TT (median (IQR); second)	16.60 (15.22, 17.90)	17.00 (15.90, 18.30)	15.70 (14.80, 17.40)	0.027
Fibrinogen (median (IQR); g/L)	4.34 (3.37, 5.22)	4.09 (2.76, 4.79)	4.56 (3.84, 5.90)	0.022
FDP (median (IQR); μg/L)	20.20 (12.00, 32.80)	20.50 (12.50, 30.10)	19.50 (11.80, 34.50)	0.928
DD‐dimer (median (IQR); mg/L)	5.58 (3.18, 9.60)	5.68 (4.24, 9.60)	5.54 (3.11, 9.16)	0.688
Platelet (median (IQR); ×10^9^/L)	172.50 (111.75, 293.25)	173.00 (102.00, 304.00)	172.00 (117.00, 284.00)	0.977

*Note*: Descriptive statistics are presented as median (IQR) for continuous characteristics and frequency (percentage, %) for categorical characteristics.

*p*‐Values were derived from Fisher's exact test for categorical characteristics and Mann–Whitney *U* test for continuous characteristics.

Abbreviations: APACHE Ⅱ, acute physiology and chronic health evaluation Ⅱ; ARDS, acute respiratory distress syndrome; AKI, acute kidney injury; APTT, activated partial thromboplastin time; BMI, body mass index; FDP, fibrinogen degradation products; IQR, interquartile range; INR, international normalized ratio; PT, prothrombin time; SIH, spontaneous intraabdominal hemorrhage; TT, thrombin time.

^a^ The characteristics, i.e., APACHE Ⅱ, showed no significant difference after propensity matching.

^b^ Age at the time of recruitment.

The median (interquartile range) age at admission was 46 (33–56) years old for the SIH‐SAP group, and 38 (30–44) years old for the non‐SIH‐SAP group (*p* = 0.007). Females constituted a smaller proportion of the SIH‐SAP group compared to the non‐SIH‐SAP group (17.8% vs. 46.7%, *p* = 0.006). The mortality rate of SIH‐SAP patients (35.6%) is extensively higher than that of non‐SIH‐SAP patients (22.2%), while no significant difference was observed. Coagulation functional tests showed notable differences between the groups. The analysis demonstrated a higher thrombin time (median seconds; interquartile range) in the SIH‐SAP group (17.00; 15.90–18.30) than the non‐SIH‐SAP group (15.70; 14.80–17.40; *p* = 0.027), and lower serum fibrinogen levels (median g/L, interquartile range) in the SIH‐SAP group (4.09; 2.76–4.79) than the non‐SIH‐SAP group (4.56; 3.84–5.90; *p* = 0.022). The prolonged thrombin time in SIH‐SAP patients indicated a tendency toward fibrinogen deficiency or dysfunction, while the lower serum fibrinogen levels, critical for blood clot formation during tissue or vascular injury, suggested a slightly impaired coagulation function.

### Whole exome sequencing and candidate variants selection

**FIGURE 2:**
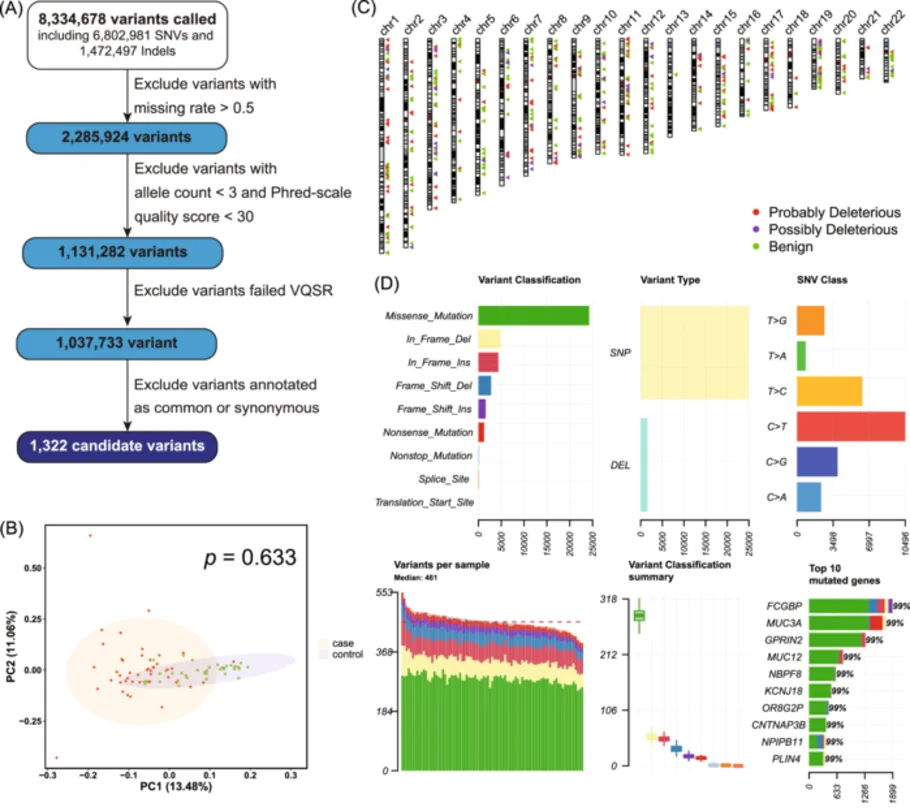
Overview of candidate variant selection and characterization. (A) Flow‐chart illustrates inclusion and exclusion of genetic variants, which resulted in the identification of 1322 exonic variant candidates. (B) Genetic principal component analysis of the included participants. (C) Distribution of predicted candidate variants categorized as deleterious or benign based on the Sorting Intolerant from Tolerant (SIFT) algorithm. Red, purple, and green triangles denote probably deleterious, possibly deleterious, and benign sites, respectively. (D) Summary characteristics of the candidate exonic variants. DEL, deletion; SNV, single nucleotide variants; SNP, single‐nucleotide polymorphism; SIFT, sorting intolerant from tolerant; VQSR, variant quality score recalibration.

Except for the sequencing data of five patients in the non‐SIH‐SAP group, which failed to meet quality requirements and thereby were excluded from the WES analysis, all remaining samples produced high‐quality WES data, with >80.0% of the quality score ≥Q30 (99.9% base call accuracy; Table S1). On average, 41 million reads per sample (range: 35–51 million) were generated, with an average read length of 150 bp. Over 99.9% of reads for each sample were on target, yielding 8,334,678 variants. Then, the variants with a missing rate >0.5, a minimal frequency of alternative alleles in the SIH‐SAP group <3, a Phred‐scaled quality value <30, or disqualified variant quality score recalibration were excluded. After quality control, a total of 1,037,733 variants (12.5%) remained for further analysis. These variants were annotated and screened for variants of interest (minor allele frequency (MAF) <1% and nonsynonymous), resulting in 1322 candidates for further analysis (Figure 2A). Principal components analysis (PCA) revealed a homogenous population structure between the SIH‐SAP patients and non‐SIH‐SAP patients (*p* = 0.633) (Figure 2B).

Of the candidate variants, nearly 22.3% (295 variants) were predicted to be “probably deleterious” or “possibly deleterious” based on Sorting Intolerant from Tolerant algorithm (SIFT) (Figure 2C), while 16.6% (220 variants) were predicted to be “probably damaging” or “possibly damaging” based on Polymorphism Phenotyping (PolyPhen). The average number of candidate variants per sample was 461, with *FCGBP* having the highest number of variants among the set of variants analysed (Figure 2D).

Comprehensive annotation of the candidate variants (Tables S2–S11) revealed that the majority were classified as missense variants (63.8%), followed by in‐frame deletion/insertions (20.0%) and frame‐shift deletion/insertions (13.9%). Many of these variants were associated with enhancer histone marks and epigenetic alterations. Notably, 367 variants were observed to be located in the CpG island sites, indicating potential DNA methylation and epigenetic alterations. In addition, gene enrichment pathway analysis using the Gene Ontology (GO) database identified 68 significant pathways (*p* < 0.05), primarily related to protein metabolism and extracellular matrix organization.

### The *FCGBP* rs1326680184 variant is associated with SIH‐SAP

**FIGURE 3:**
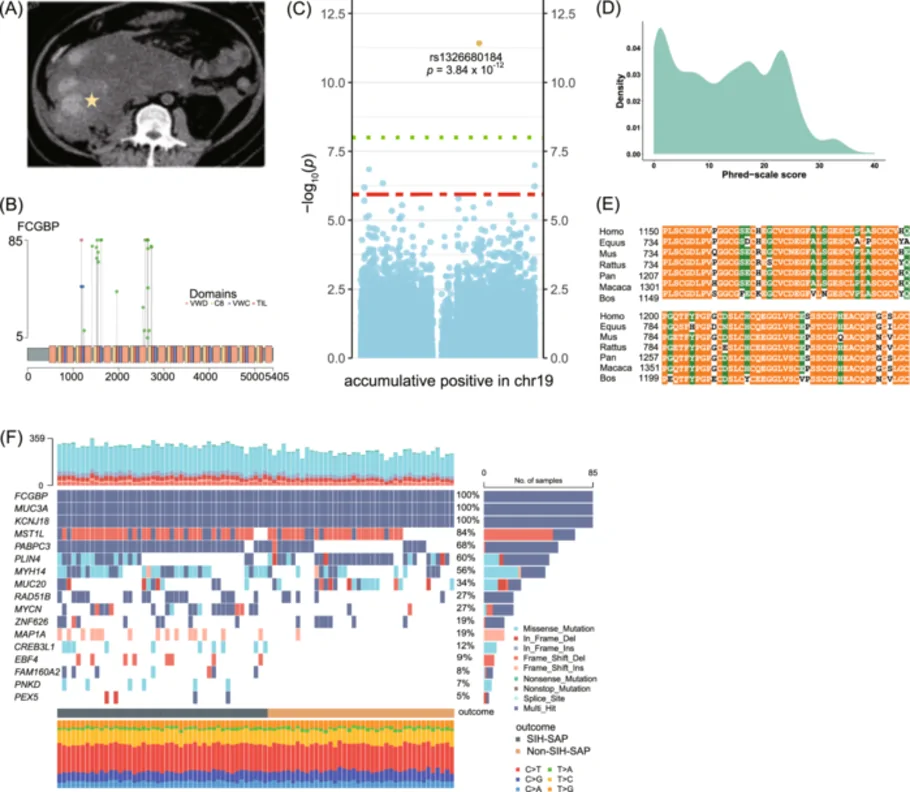
Bioinformatics analysis identify *FCGBP* and its variant to be associated with SIH development. (A) Example of computed tomography for SIH in an SAP patient. The yellow pentagram indicates the focal lesion of SIH. (B) Distribution of *FCGBP* variants among SIH‐SAP patients. A frame‐shift deletion is symbolized by a blue circle, whereas a green circle denotes an altered amino acid. (C) Manhattan plot of GWAS based on exome variants in chromosome 19. The *x*‐axis is chromosomal position, and the *y*‐axis is –log_10_(*p*‐value of GWAS). The rs1326680184 was used and denoted to represent the six identified *FCGBP* variants. (D) Distribution of CADD score of the 1322 candidate variants. (E) Protein sequence alignment of FCGBP across multiple species: *Homo sapiens*, *Equus caballus*, *Mus musculus*, *Rattus norvegicus*, *Pan troglodytes*, *Macaca mulatta*, and *Bos taurus*. The alignment shows that the screened residues of FCGBP are highly conserved across species and isoforms. Residues that are absolutely conserved and highly conserved are highlighted in orange and green, respectively. (F) Gene‐based variants in SAP patients. The matrix in the middle panel indicates each column representing an individual patient and each row representing a gene. The bar plot at the top shows the number of variants in each individual, while the bar plot on the right shows the percentage of individuals with variants in each gene. The bottom plot shows the distribution of 6 types of variants per individual. AP, acute pancreatitis patients; CADD, combined annotation dependent depletion; FCGBP/Fcgbp, Fc fragment of immunoglobulin G binding protein; GWAS, genome‐wide association study; LPS, lipopolysaccharides; MAF, minor allele frequency; PBS, phosphate buffer saline; RNAi‐AAV, adeno‐associated virus packaged with small hairpin RNA; SIH, spontaneous intraabdominal hemorrhage; SAP, severe acute pancreatitis.

Among the 1322 candidate variants, allele distribution between the SIH‐SAP and primary non‐SIH‐SAP groups was analysed to identify potential pathogenic variants with significantly different alternative allele frequencies (*p*_adj_ < 0.05). This analysis identified a frame‐shift deletion (rs1326680184, deletion of base TC) located in *FCGBP* (RefSeq NM_003890) as strongly associated with SIH development in SAP patients. Notably, this variant was significantly more prevalent in SIH‐SAP patients (36/45; 80.0%) compared to non‐SIH‐SAP patients (11/40; 27.5%; Figure 3A,B), indicating its potential role in SIH susceptibility.

To validate these findings, an independent secondary control group consisting of 510 AP patients from a prospectively registered AP cohort in our centre was included for further analysis. Consistent with the initial results, the *FCGBP* rs1326680184 variant remained significantly associated with SIH, with a higher carrier frequency in SIH‐SAP patients (36/45; 80.0%) compared to non‐SIH‐AP patients (192/510; 37.6%; *p*_fdr_ < 0.001). Importantly, this association was consistent across different severities of AP, with similar carrier prevalence in severe or critical AP patients (38.9%) and mild or moderate AP (36.8%, *p* > 0.05), suggesting that the effect of rs1326680184 on SIH is conserved, irrespective of AP severity. Furthermore, no additional variants were found to be associated with SIH in sensitivity analysis, which included all 1,037,733 variants passing quality control.

To address potential multiple testing bias in this relatively small sample size, we employed a targeted genome wide association study focusing on chromosome 19, comparing 45 SIH‐SAP patients to 40 non‐SIH‐SAP patients while controlling for age and gender. The rs1326680184 was consistently associated with the development of SIH (*p*_adj_ = 3.84 × 10^–12^; Figure 3C and Figure S1). Moreover, an exome‐wide association analysis across all chromosomes, revealed multiple subtle signals with *p*_adj_ < 5 × 10^–6^ (Figure S2).

Genomic annotation further characterized that rs1326680184 is located at chromosome 19:39906058‐39906060 within the exonic region of the *FCGBP* gene and is a frame‐shit deletion. Using the combined annotation‐dependent depletion score [14], rs1326680184 was predicted to be deleterious with a scaled C‐score of 20.7, indicating a likely disruptive effect (Figure 3D). Additionally, conservation analysis suggested that the affected region of *FCGBP* is highly conserved across species and isoforms (Figure 3E).

Lastly, a sequence kernel association test (SKAT) analysis [15, 16] identified 17 genes suggestively associated with SIH in SAP patients (*p* < 0.01) (Table S12). Among these, *FCGBP* ranked as the top hit (*p* = 1.310 × 10^–7^ in SKAT; Figure 3F), further supporting its pivotal role in SIH pathogenesis.

### ELISA validation of *FCGBP* expression associated with rs1326680184 in AP patients

**FIGURE 4:**
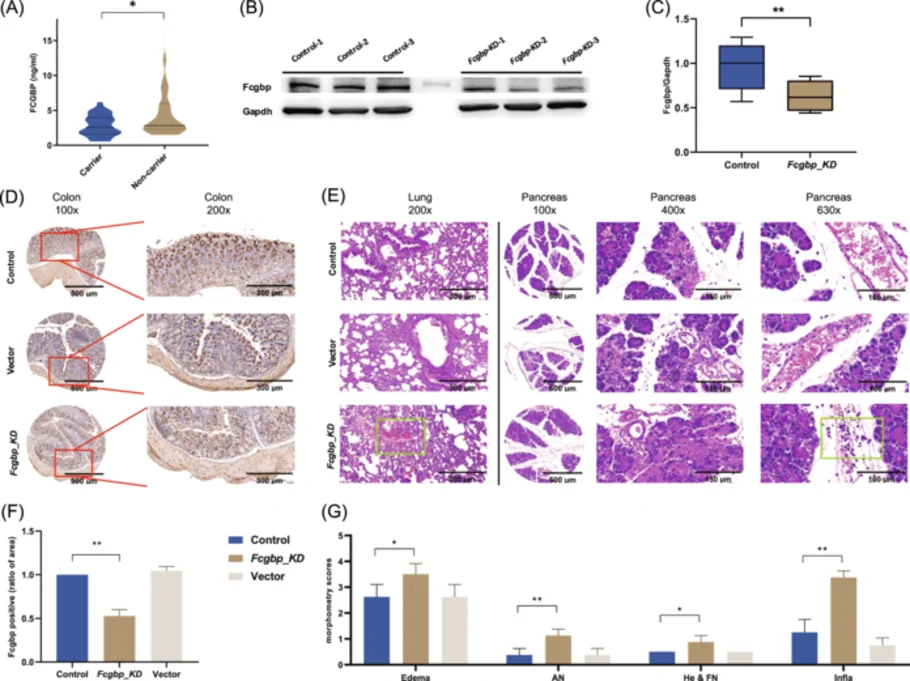
*FCGBP* variant (i.e., rs1326680184) relates to lower expression of *FCGBP* in patients, and *Fcgbp*‐KD exacerbates the severity and lung injury of AP in mice. (A) Violin plot shows the concentrations of FCGBP protein in the peripheral blood of AP patients, comparing rs1326680181 carriers and non‐carriers (*n =* 27 in each group), * indicates *p* < 0.05 by Student's *t*‐test with Welch's correction. (B) Western‐blot analysis of Fcgbp protein in colon tissue of mice that underwent *Fcgbp* knockdown (*Fcgbp*‐KD) compared to normal healthy mice. (C) Quantification of western‐blot presents the ratio of Fcgbp/Gapdh (*n* = 6 in each group), ** indicates *p* < 0.01 by the Man–Whitney test. (D) Immunohistochemical analysis of *Fcgbp* expression in mice colon tissue. AAV‐RNAi targeting *Fcgbp* inhibits the expression of *Fcgbp*, compared with AAV empty vector and PBS controls (*n* = 10 in each group). (E) Morphological examination of lung and pancreas tissue (H&E staining). Results show edema, acinar necrosis, hemorrhage, and inflammation. Cellular edema and damage, and neutrophils infiltration are significantly more severe in *Fcgbp*‐KD mice. Leakage of red blood cells is present in *Fcgbp*‐KD mice but not in the AAV empty vector injected mice and PBS controls. (F) Quantification of *Fcgbp* expression in the stained area using ImageJ, with the control group as reference. ** indicates *p* < 0.01 by one‐way ANOVA with post hoc tests. Error bars are shown as SEM. (G) Quantification of severity based on the pathological scoring scale. * indicates *p* < 0.05, *** indicates *p* < 0.001 by one‐way ANOVA with post hoc tests. Error bars are shown as SEM (*n* = 10 in each group). AAV, adeno‐associated virus; ANOVA, analysis of variance; AN, acinar cell necrosis; FCGBP/Fcgbp, Fc fragment of immunoglobulin G binding protein; KD, knockdown; GAPDH, glyceraldehyde‐3‐phosphate dehydrogenase; He & FN, hemorrhage and fat necrosis; Infla, inflammation; PBS, phosphate buffer saline; RNAi‐AAV, adeno‐associated virus packaged with small hairpin RNA; SEM, standard error of mean.

Enzyme‐linked immunosorbent assay (ELISA) was conducted to quantify FCGBP protein levels in the peripheral blood of randomly selected AP patients, stratified by the presence (*n* = 27) or absence (*n* = 27) of rs1326680184. The results showed that AP patients with rs1326680184 had lower FCGBP levels compared to those without rs1326680184 (mean of 2.86 ng/mL vs. 4.66 ng/mL, *p* = 0.035, Figure 4A). To assess whether reduced FCGBP levels predispose to SIH, FCGBP protein levels were compared between SIH (*n* = 30) and non‐SIH patients (*n* = 30). The results showed that AP patients with SIH had significantly lower levels of FCGBP compared to non‐SIH‐SAP patients (*p* < 0.001, Figure S3). These findings demonstrated a consistent relationship that rs1326680184 is associated with reduced *FCGBP* expression level, which is linked to an increased predisposition to SIH in AP patients.

### *Fcgbp* knockdown exacerbates the severity and multiorgan injury of AP in mice

Building upon the observed link between reduced FCGBP levels and predisposition to SIH, we further investigated the functional consequences of FCGBP deficiency using a mouse model with *Fcgbp‐*knockdown (*Fcgbp*‐KD). The knockdown was achieved through intravenous injection of adeno‐associated virus harbouring short harpin RNA targeting *Fcgbp*. Western‐blot analysis of colon tissue identified as the most enriched site of Fcgbp [17], revealed an average decrease of 41.5% in *Fcgbp* expression in *Fcgbp*‐KD mice compared to the control mice (*p* = 0.002, Figure 4B,C). This reduction in *Fcgbp* expression was further validated through immunohistochemistry (IHC) staining in colon tissue (Figure 4D,F), as well as lung tissue (Figure S4) and vessel tissue (Figure S5).

To evaluate the impact of *Fcgbp* knockdown, the *Fcgbp*‐KD mice, together with control groups injected with either AAV vector or phosphate‐buffered saline, were subjected to an SAP model generation through intraperitoneal injection of caerulein and lipopolysaccharide. Based on the pathomorphological examination, the *Fcgbp*‐KD mice presented more severe tissue damage in the pancreas and lungs compared to control mice (Figure 4E). Specifically, more pronounced edema and destruction of alveolar structures were observed in the lungs in mice of *Fcgbp*‐KD. In the pancreas, in line with previously established morphometric criteria [18] for assessing AP severity (Table S13), *Fcgbp*‐KD mice exhibited significantly more severe edema (3.50 vs. 2.63, *p* = 0.032), acinar cell necrosis (1.13 vs. 0.38, *p* = 0.005) and inflammation (3.38 vs. 1.25, *p* < 0.001) compared to control mice. Additionally, *Fcgbp*‐KD mice exhibited erythrocytes leakage into the interlobular space of the pancreas and pulmonary alveoli, which was absent in control mice, further implicating Fcgbp deficiency in heightened vascular injury and hemorrhage (Figure 4E,G). These results suggest that Fcgbp deficiency exacerbates pancreas damage during AP.

**FIGURE 5:**
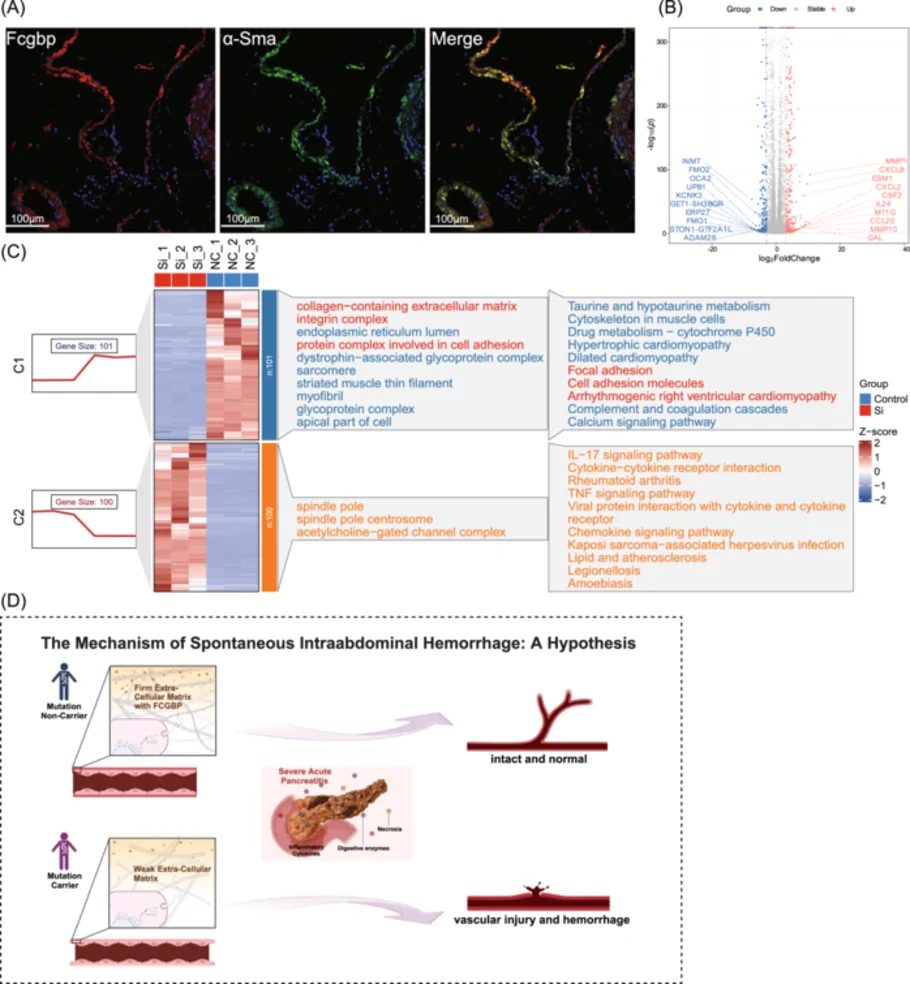
Vascular fibroblasts produce FCGBP and function as stabilizers of the vascular wall. (A) Representative immunofluorescence images show the co‐localization of Fcgbp and α‐Sma in vascular tissue. (B) volcano plot of differentially expressed genes (DEGs) in *FCGBP*‐knockdown fibroblasts. (C) Heatmap of the top 100 upregulated and top 101 downregulated DEGs in *FCGBP* knockdown fibroblasts, with enrichment of corresponding gene sets presented on the side. (D) Hypothetic mechanism of how the *FCGBP* variant (rs1326680184) affecting the predisposition of SIH in SAP: rs1326680184 carrier suffered a weak extracellular matrix compared with non‐carrier, resulting in a higher risk of vascular injury and hemorrhage under the impact of severe acute pancreatitis. FCGBP/Fcgbp, Fc fragment of immunoglobulin G binding protein; SIH, spontaneous intraabdominal hemorrhage; SAP, severe acute pancreatitis.

To further explore the protective role of FCGBP against SIH, we conducted immunofluorescence analysis on blood vessel of mice. The results indicated that FCGBP is primarily produced by fibroblasts, as indicated by α‐Sma staining (Figure 5A). Next, comprehensive mRNA expression profiling of the *FCGBP*‐knockdown and wild‐type human vascular fibroblasts identified 456 upregulated and 309 downregulated genes (Figure 5B, Figures S6, S7). Subsequent enrichment analysis of the downregulated genes showed significant enrichments in the GO terms of “extracellular matrix,” “collagen‐containing extracellular matrix,” and “external encapsulating structure” (Figure 5C). Additionally, we analysed published single‐cell RNA‐sequencing data from pancreatic cancer samples (https://www.fibroxplorer.com/) and demonstrated that *FCGBP* was specifically expressed in fibroblast subtypes characterized by *COL3A1* or *PI16* expression, both of which are highly associated with extracellular matrix (ECM) remodelling and vascular fibrosis (Figure S8A, S8B). A separate sing‐cell data set including samples from thoracic aorta aneurysm and normal thoracic aorta tissues (GEO accession number GSE155468) similarly demonstrated that ECM‐related genes, such as *COL1A1*, *COL3A1*, and *TIMP1*, were generally downregulated in aneurysmal samples, alongside reduced *FCGBP* expression (Figure S8C, S8D).

### Association between rs1326680184 and clinical characteristics

**FIGURE 6:**
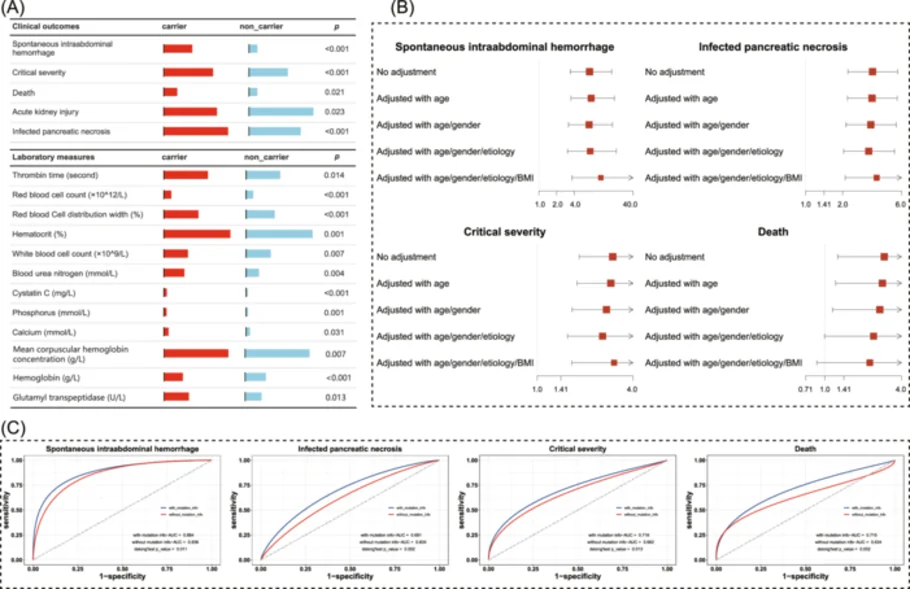
Associations between *FCGBP* variant (i.e., rs1326680184) and clinical characteristics. (A) Comparison of clinical characteristics at admission between rs1326680184 variant carriers and non‐carriers. (B) Forest plots of rs1326680184 variant with the risk of SIH, critical severity of SAP, infected pancreatic necrosis, and death. The odds ratios (ORs) were shown by solid square and horizontal lines representing the 95% confidence intervals; the adjustments were performed with age, gender, BMI, and etiology of AP. (C) Receiver operating characteristic curve (ROC) and diagnostic ability of demographic factors and rs1326680184 variant in relation to multiple clinical characteristics. The statistical comparison was conducted using DeLong's test, in which the *p* < 0.05 was considered as statistical significance. AP, acute pancreatitis; BMI, body mass index; ORs, odds ratios; ROC, receiver operating characteristic curve.

Out of 600 included participants, 327 AP patients were eligible for further assessment of the association between rs1326680184 and clinical characteristics, based on the accessibility of complete genotyping, clinical outcomes, and laboratory measures. Among these patients, 144 (44.0%) were rs1326680184 carriers, while 183 (56.0%) were non‐carriers. The two groups showed no difference in age, gender, and BMI. However, the rs1326680184 carriers showed a significantly prolonged thrombin time compared to non‐carriers (mean of 19.24 s vs. 16.97 s; *p* = 0.010), indicating that rs1326680184 may adversely affect coagulation function. Additionally, the rs1326680184 carriers had worst clinical outcomes, with a higher rate of mortality (11.8% vs. 4.4%; *p* = 0.021), acute kidney injury (46.5% vs. 33.5%; *p* = 0.023), IPN (56.2% vs. 26.9%; *p* < 0.001), and critical form of AP (43.1% vs. 20.2%; *p* < 0.001) (Figure 6A; Table S14). These findings support the aforementioned observations that rs1326680184 may increase the predisposition to hemorrhage, thereby leading to poorer clinical outcomes in AP patients. Moreover, the rs1326680184 carriers tended to have hematological abnormalities, including significantly lower red blood cell counts (mean of 3.22 × 10^9^/L vs. 3.57 × 10^9^/L; *p* < 0.001), hemoglobin concentration (mean of 95.36 g/L vs. 107.30 g/L; *p* < 0.001) and hematocrit levels (mean of 29.0% vs. 30.0%; *p* = 0.001) (Table S15). These results provide further evidence that phenotypes related to SIH are more pronounced in rs1326680184 carriers compared to non‐carriers.

To evaluate the clinical utility of rs1326680184, logistic regression analysis was performed, comparing carriers to non‐carriers. The results revealed the rs1326680184 carriers were at significantly higher risk of developing SIH (OR = 11.35; 95%CI: 3.66‐45.81; *p*_adj_ < 0.001), severe AP (OR = 3.04; 95%CI: 1.65‐5.71; *p*_adj_ < 0.001), and IPN (OR = 3.77; 95%CI: 2.09–6.93; *p*_adj_ < 0.001) (Figure 6B). These findings were validated using multiple models adjusted for different demographic factors. When compared to the model based on conventional demographic factors (i.e., age, gender, and etiology of AP), the model incorporating rs1326680184 achieved better predictive performance. The inclusion of rs1326680184 improved the area under receiver operating curve (AUROC)) for diagnosing clinical outcomes as follows: SIH (0.84 vs. 0.88, *p* = 0.011), IPN (0.61 vs. 0.69, *p* = 0.002), the severity of AP (0.66 vs. 0.7, *p* = 0.013), death (0.63 vs. 0.72, *p* = 0.052). These results suggest *FCGBP* variant could serve as a robust risk indicator, enhancing the prediction and diagnosis of complications and prognosis of AP with stable diagnostic performance (Figure 6C).

## 3 DISCUSSION

This study, leveraging the largest‐scale WES data of AP patients to date, presents novel insights into the genetic architecture, biological mechanisms, and clinical translations of SIH, a life‐threatening complication. These findings provide significant potential for improving critical care in AP.

By single‐variant and gene‐based analysis, we identified a genetic variant located in the *FCGBP* gene that is enriched in patients with SAP and SIH. In silico prediction models suggest that this variant has a deleterious effect, altering FCGBP protein levels and increasing predisposition to SIH. This was associated with adverse laboratory measures and poorer prognosis in AP. Validated through in vivo animal model confirmed that *Fcgbp* knockdown mice showed an increased severity of AP and higher SIH predisposition, accompanied by reduced *Fcgbp* expression in multiple organs.

The functions and regulatory mechanisms of *FCGBP* remain insufficiently studied. Previous studies, such as Harada et al. [17], have found that human *FCGBP* is widely expressed in systemic mucosa and exhibits a mucin‐like structure, likely involved in antigen trapping and playing a pivotal role in the immune and inflammatory process. Nevertheless, robust evidence on how FCGBP functions in human health, both in the immune system and nonimmune systems, is lacking. While most studies focused on *FCGBP* in neoplastic diseases [19, 20, 21, 22], recent studies have indicated its involvement in vascular disorders. For example, Gäbel et al. linked the altered *FCGBP* expression with angiogenesis and abdominal aortic aneurysms progression [23], while Zhang et al. demonstrated that a *FCGBP* variant was associated with the development of brain arteriovenous malformation [24].

For the first time, using WES combined with both variants and gene‐set analysis, we uncovered and validated a *FCGBP* variant accompanied by the development of SIH in SAP patients. Specifically, we identified a frame‐shift deletion in *FCGBP* (rs1326680184) that potentially diminishes protein expression. Based on this, we hypothesized that rs1326680184 carriers have increasing susceptibility and predisposition to vascular injury and hemorrhage in severe inflammatory conditions such as SAP (Figure 5D). To verify this hypothesis, we used immunofluorescence analysis on vascular tissue, identifying vascular fibroblasts as key producers of Fcgbp. Further mRNA expression profiling experiment using cultured human vascular fibroblasts, in which *FCGBP* was knocked down, revealed that differentially regulated genes were enriched in pathways related to vascular stability. These findings support our hypothesis that reduced *FCGBP* expression could destabilize the vascular wall, and lead to predisposition to vascular injury in SAP. However, the lack of protein structure of FCGBP limited our exploration to elucidate its mechanistic pathways in SIH, emphasizing the need for further molecular studies.

To explore the translational significance of the above findings, we developed a clinical classification model that incorporated rs1326680184. This model demonstrated improved diagnostic capacity for SIH, as well as improved predictions of IPN, disease severity, and mortality of AP. Thus, FCGBP alteration could serve as both a diagnostic biomarker and a therapeutic target for SAP and other critical diseases, enhancing more precise prognostic assessments and tailored treatments.

To our knowledge, this study represents the first attempt to discover novel genetic associations using WES in a population of patients with AP. Here, we identified strong associations between a *FCGBP* exonic variant (rs1326680184) and SIH, with implications for other complications, the severity, and mortality of AP. These findings could guide the development of urgently needed therapies to prevent life‐threatening complications in the early phase of AP and improve patient outcomes.

This study has several limitations. First, although our cohort consisted of a rare population of SAP patients with SIH, the sample size for the genetic study was relatively small. Nevertheless, through applying the multi‐stage analysis and experimental validations, we achieved sufficient statistical power to detect meaningful differences. Second, while the cohort included AP patients from multiple areas across China, external validation in independent populations with different ethnicities is required to confirm these findings. Third, the retrospective design introduces the potential for residual bias, although stringent clinical assessment and precise laboratory measures minimized this risk. The retrospective nature also impacted sample quality, leading certain sequencing limitations during WES analysis. Additionally, WES has inherent constraints, such as difficulty in accurately capturing structural variants (e.g., copy number variants). Fourth, although the *Fcgbp*‐KD mouse provided valuable in vivo evidence, the use of *Fcgbp* knockout (*Fcgbp^–/–^*) mice could have offered additional evidence. However, the ethical consideration of animal suffering and uncertainty about *FCGBP* function justified our decision. Finally, the unknown FCGBP protein structure impeded detailed mechanistic exploration, warranting future structural biological research. Despite these limitations, this study significantly advances our understanding of SIH in AP and provides a foundation for further functional analysis and therapeutic developments.

## 4 METHODS

### Participant enrolment

All the patients related to AP and admitted to Jinling Hospital, a tertiary‐level referral centre in Nanjing, China, from January 1, 2015 to December 31, 2020, were retrospectively reviewed for eligibility in this study. All participants were informed about the purpose during the admission interview and provided with consent before enrolment. The study design and conduct complied with all relevant regulations regarding the use of human participants and was in accordance with the criteria set by the Declaration of Helsinki. The study protocol was approved by the Ethics Committee of Jinling Hospital, Nanjing, China (ref. No. 2021NZKY‐042‐01).

To define SAP complicated with SIH (i.e., SIH‐SAP), all the participants were assessed according to stringent inclusion and exclusion criteria. The inclusion criteria included: i) diagnosed as AP according to the revised Atlanta criteria (RAC) [25], ii) attributed as severe or critical AP according to DBC guideline [13], iii) presence of intraabdominal hemorrhage on contrast‐enhancing computed tomography without prior invasive intervention. The exclusion criteria included: (i) participants who had a history of hemophilia, idiopathic thrombocytopenic purpura, abdominal aorta aneurysm, or other hemorrhage‐associated disorders, (ii) who received anticoagulation therapies within a week before admission.

The primary control group of SAP patients without SIH (non‐SIH‐SAP) was selected using a 1:1 ratio PSM based on the severity of AP. The severity was quantified based on Acute Physiology and Chronic Health Evaluation Ⅱ (APACHE Ⅱ) score and classified as mild, moderate, severe, or critical according to DBC guidelines [13]. Additionally, to further validate the generalizability and stability of identified signals in SIH‐SAP patients, a secondary control group was established consisting of AP patients without SIH (i.e., non‐SIH‐AP). Since 4 patients refused to sign the informed consent form for using their blood to conduct WES, a total of 45 SIH‐SAP patients, 45 non‐SIH‐SAP patients, and 510 non‐SIH‐AP patients were included in the current study. Clinical and anthropometric information was obtained from medical interviews, physical examinations, and laboratory assessments at admission.

### Whole exome sequencing

Peripheral blood samples were collected within 1 day after admission and stored at –80°C. Genomic DNA of all participants was extracted from stored peripheral blood using the TIANGEN kit (Beijing, China), following the manufacturer's protocols. Whole‐exome libraries were generated according to the manufacturer's protocols, with the enrichment of exon‐coding regions using Agilent's V6 capture reagent (Agilent Technologies). Paired‐end (2 × 150 base pairs) sequencing was performed on Illumina NovaSeq. 6000 System (Illumina) (please see details in Supplementary methods).

#### Variant‐based analysis

After quality control and variant screening (as described in the supplementary methods), a total of 1322 candidate variants were selected for further analysis based on their potential pathogenic implications. These included uncommon variants (MAF < 0.01) and variants lacking population allele frequency annotations. Candidate variants with different allele frequency between the case group (SIH‐SAP, *n* = 45) and the primary control group (non‐SIH‐SAP, *n* = 40) were identified as probable pathogenic variants using *Fisher's* exact test (R package “*stats*”) with FDR correction via Benjamini–Hochberg method (*p*_fdr_ < 0.05).

To validate the identified exonic variants, the secondary control group, consisting of 510 non‐SIH‐AP patients, was analysed. *Fisher's* exact test was performed to compare the allele frequency distribution between this control group and the SIH‐SAP group (*p*_fdr_ < 0.05). As a sensitivity analysis, all the 1,037,733 variants that passed quality control also underwent variant‐based analysis following the methodology described above.

#### Targeted genome‐wide association analysis

Based on the identified signals, a chromosome‐19‐specific analysis was performed to minimize the effects of genome‐wide multiple testing. High‐quality variants from the SIH‐SAP group and non‐SIH‐SAP group underwent stringent quality control measures and were subsequently merged. Variants from both groups were filtered using PLINK (version 1.9.0) [26] to ensure reliability. A total of 43,486 variants located on chromosome 19 were deemed eligible for analysis.

Samples were excluded if they failed genotyping in more than 40% of variants. Variants were excluded if they met any of the following criteria: (i) call rate <60%; (ii) MAF <0.01; (iii) significant deviation from Hardy–Weinberg equilibrium with *p* < 1 × 10^–8^ [27]. The significance threshold was set at an exome‐wide level of *p* < 5 × 10^–6^ (5 × 10^–8^/1%, as exome accounts for approximately 1% of human genome). PLINK was used to perform this chromosome‐19‐specific targeted genome‐wide association analysis, controlling for age (in years) and gender (male or female) as covariates.

Additionally, an exome‐wide association analysis was performed with variants detected across all chromosomes to account for any unexpected signals, applying the same statistical models and criteria used in the chromosome‐19‐specific analysis.

#### Gene‐based analysis

Variants with MAF < 0.01 or lacking allele frequency information in genome reference databases (i.e., Exome Aggregation Consortium and 1000 Genomes) and predicted to be deleterious were subjected to gene annotation. Sequence kernel association test (SKAT) [15, 16], a gene‐based analysis, was employed to explore associations between genes containing at least two exonic variants, such as *FCGBP*, and the development of SIH in all included participants. Since not all variants defined as “uncommon” have a MAF < 1% in the SIH‐SAP group, a modified definition was applied. Variants were categorized as “uncommon” if $\text{MAF}\;\leq \;1∕\sqrt{2n}$, and as “common” variants if $\text{MAF}\;>\;1∕\sqrt{2n}$, where *n* represents the total sample size [16]. The combined effects of uncommon and common exonic variants on SIH were assessed by using the R package “*SKAT*.”

### Animal experiments

All animal experiment procedures followed the Chinese Animal Welfare Act and the Guidance for Animal Experimentation of Southeast University (Approval no. 2022DZGKJDWLS‐0059). Male ICR mice aged 8 weeks were obtained from Ziyuan Laboratory Animal Technology Co., Ltd. Animals were housed in specific pathogen‐free facilities under a 12‐h light‐dark cycle, with free access to standard food and water.

#### Knockdown of *Fcgbp*

*Fcgbp* was silenced using an adeno‐associated virus (AAV) vector delivery system. An AAV vector harboring a short hairpin RNA (shRNA) targeting *Fcgbp* (RefSeq NM_001122603.1) (AAV‐RNAi) was provided by Gene Chem Co., Shanghai, China. For *Fcgbp*‐target AAV‐RNAi packaging, the designed shRNA duplexes (Table S16) were cloned into the vector. The titer of AAV was 3.36 × 10^13^ viral genome copies per mL, and each mouse received a total of 5.00 × 10^11^ viral genome copies via intravenous injection.

#### In vivo acute pancreatitis mice model

The SAP mouse model was induced by intraperitoneal injection of caerulein (50 μg/kg, administrated seven times at 1‐h intervals), followed by a single dose of lipopolysaccharide (5 mg/kg) administered 1 h after the final caerulein injection [28]. Pathological examination of pancreas tissue from SAP mice and normal mice is presented in Figure S9. Blood assay results for TNF‐α, IL‐6, and MCP1 are provided in Figure S10. *Fcgbp*‐KD mice, together with mice injected with either the AAV vector or PBS, were subjected to SAP induction on the 30th day postinjection of AAV or PBS injection (*n* = 10 per group). At 24 h after SAP induction, the mice were anesthetized, and tissues of lung, pancreas, colon, and vessel were harvested. Mice were killed with measures taken to minimize suffering.

#### Immunohistochemistry

Immunohistochemistry (IHC) was performed to assess *Fcgbp* expression in lung, colon, and blood vessel tissues obtained from the killed mice. Briefly, 5‐μm‐thick cryosections of quick‐frozen lung, colon, and vessel tissues were fixed in acetone, quenched with 3% H_2_O_2_, and blocked with goat serum. After washing with PBS, the sections were treated with anti‐*Fcgbp* primary antibody (bs‐13168R, Bioss) for 2 h. The sections were then incubated with horseradish‐peroxidase‐conjugated secondary antibody for an hour. Colour development was achieved using 3,3’‐diaminobenzidine tetra hydrochlorides as the peroxidase substrate. Fcgbp‐positive areas were quantified using ImageJ. Differences of positive staining among three groups (control, AAV‐RNAi, and AAV vector groups) were compared using one‐way ANOVA with post‐hoc tests to evaluate statistical significance.

#### Immunofluorescence

Immunofluorescence was conducted to detect Fcgbp, Cd31, α‐Sma, and Vegf expression in vessel tissues obtained from killed mice. The procedure followed the same steps as IHC up to secondary antibody incubation. After three PBS washes, the sections were treated with fluorescent dye to visualize the target proteins.

#### Morphological examination

Fresh pancreatic and pulmonary tissue samples were fixed with 4% paraformaldehyde overnight. After fixation, the samples were washed with running water for 2 h, dehydrated through an ethanol gradient, embedded in paraffin, and sectioned into 5‐μm‐thick slices. The sections were dewaxed in xylene, rehydrated using graded ethanol solutions (100%, 95%, 80%, and 70%), and stained with hematoxylin and eosin. Morphological examination included edema area and grade, acinar cell necrosis, inflammatory reaction, adipose necrosis, and hemorrhage. These parameters were used to determine the severity of AP in each group of mice (*n* = 10 per group). Evaluations were conducted independently by two blinded investigators using previously reported morphometry methods [18]. Differences in severity scores among the three groups were evaluated using one‐way ANOVA with post‐hoc tests to determine statistical significance.

#### Cell lines and cell culture

The human vascular fibroblast cell was obtained from Wuhan Sunncell Biotech Co., Ltd. and cultured in a specific complete culture medium provided by Wuhan Sunncell Biotech Co., Ltd. (SNPM‐H362). The cells were maintained in a humidified incubator at 37°C with 5% CO_2,_ in a medium supplemented with 10% fetal bovine serum (Gibco) and 1% penicillin and streptomycin (Gibco).

#### Construction of knockdown cell

Cells designated for knockdown construction were thoroughly digested and plated in six‐well plates at a confluency of 70%–80%. The cells were then transfected with either *FCGBP*‐targeting siRNAs or control siRNA and cultured for 2 days before further experiments. The efficiency of the knockdown was assessed by qRT‐PCR and western blot analysis.

#### RNA‐Seq and bioinformatics analysis

Total cellular RNA was extracted from both the knockdown fibroblasts and the control cells, with residual DNA removed using DNase I (Roche Diagnostics). RNA‐seq libraries were prepared using the TruSeq RNA Library Prep Kit and sequenced on the Illumina NextSeq. 500 platform. The resulting sequencing data were processed to compute transcript FPKMs. Pathway analysis was performed using the GO (http://geneontology.org/) and KEGG (https://www.genome.jp/kegg/) databases.

### Investigation of identified variant in relation to clinical characteristics

AP patients in the case group, primary control group, and secondary control group were pooled (*n* = 600) irrespective of disease severity and the presence of SIH. These patients were then re‐categorized according to the genetic status of the identified variant as carriers or non‐carriers. To examine the association between the identified variant and clinical characteristics, analysis were conducted by comparing clinical features between variant carriers to non‐carriers. The comparisons involved evaluating the distribution of clinical characteristics and assessing their associations with the identified variant using a standard logistic regression adjusted for age (in years, continuous), gender (male or female), BMI (Kg/m^2^, continuous), and etiology of AP (biliary, hypertriglyceridemia, and others). Blood test parameters were obtained from the first blood draw upon hospital admission.

The receiver operating characteristic curve (ROC) (R package “*pROC*”), [29] and DeLong's test [30] for comparison of area under the ROC curve (AUROC) were employed to assess whether the identified variant increases the diagnostic ability for a series of clinical outcomes, including SIH, in addition to demographic and clinical factors.

### Statistical analysis

Descriptive statistics are presented as median (interquartile range) for continuous variables and frequency (percentage, %) for categorical variables. Demographic and clinical characteristics between different participant groups were compared using the Wilcoxon rank‐sum test for continuous variables and *Fisher's* exact test for categorical variables. All the statistical analysis were performed using R software 4.1.0 (R Core Team. R Foundation for Statistical Computing. Vienna, Austria. 2021. https://www.R-project.org) [31].

#### Statistical power and multiple hypothesis testing

To minimize the occurrence of false discoveries, the current study implemented a series of measures: (i) genetic variants with relatively low reliability were eliminated based on quality indicators, including missing rate, quality score, and VQSR; (ii) a filtration strategy was applied to restrict the number of variants tested, excluding common and synonymous variants; (iii) FDR correction was used to adjust for multiple testing when comparing allele frequency distribution among the included candidate variants; (iv) to ensure the reliability and reproducibility of results, the analysis were replicated with a secondary control group of non‐SIH‐AP patients to verify the consistency of the identified genetic signals.

## AUTHOR CONTRIBUTIONS

**Qiu‐Yi Tang**: Conceptualization; methodology, formal analysis; data curation; writing—original draft. **Yue—Peng Hu**: Resources; investigation; validation; data curation. **Qi Yang**: Resources. **Jing Zhou**: Writing—review & editing. **Jing—Zhu Zhang**: Writing—review & editing. **Jie Yang**: Writing—review & editing. **Hai—Bin Hao**: Validation; investigation; writing—review & editing. **Gang Li**: Writing—review & editing. **Bai—Qiang Li**: Writing—review & editing. **Lu Ke**: Supervision; writing—review & editing. **Zhi—Hui Tong**: Supervision; writing—review & editing. **Yu—Xiu Liu**: Supervision; writing—review & editing. **Evan Yi—Wen Yu**: Supervision; methodology; project administration; funding acquisition; writing—review & editing. **Wei—Qin Li**: Conceptualization; supervision; project administration; funding acquisition.

## CONFLICT OF INTEREST STATEMENT

The authors declare no conflicts of interest.

## 1 ETHICS STATEMENT

All participants were informed about the purpose of the study during the admission interview, with consent before enrolling in the study. The study design and conduction complied with all relevant regulations regarding the human participants and was in accordance with the criteria set by the Declaration of Helsinki. The study's protocol was approved by the Ethics Committee of Jinling Hospital, Nanjing, China (ref. No. 2021NZKY‐042‐01). All animal experiment procedures followed the Chinese Animal Welfare Act, the Guidance for Animal Experimentation of Southeast University. Efforts were made to minimize the number of animals used and reduce their suffering (ref. No. 2022DZGKJDWLS‐0059).

## Supporting information


**Figure S1.** Quantile‐quantile plot for the GWAS analysis of WES and SIH.
**Figure S2.** Manhattan plot of genome‐wide association analysis for exonic variants in all autosomal chromosomes.
**Figure S3.** Assay of serum FCGBP in patients with or without SIH.
**Figure S4.** Immunohistochemical analysis of *Fcgbp* expression in lung tissue in mice.
**Figure S5.** Immunohistochemical analysis of *Fcgbp* expression in vessel tissue in mice.
**Figure S6.** Quantitative PCR analysis of *FCGBP* mRNA level in knockdown fibroblasts and wild‐type control.
**Figure S7.** Relative expression levels of top down‐regulated genes and ECM‐related genes from RNA‐seq, assayed by qPCR.
**Figure S8.** Single‐cell RNA‐sequencing analysis of fibroblasts.
**Figure S9.** Pathology examination of lung and pancreas tissue of mice.
**Figure S10.** Assays of blood MCP1, TNFα, and IL‐6 from mice induced as AP and normal healthy mice.

**Table S1.** Quality control measures and summary sequence statistics of whole exome sequencing.
**Table S2.** Genomic coordinates of candidate variants and allele frequencies retrieved from 1000 Genome Project.
**Table S3.** Effects of candidate variants on protein function based on combined annotation dependent depletion (CADD) score.
**Table S4.** Effects of candidate variants on protein function based on Polymorphism Phenotyping (PolyPhen).
**Table S5.** Effects of candidate variants on protein function based on Sorting Intolerant from Tolerant algorithm (SIFT).
**Table S6.** Associations between identified candidate variants and clinical phenotypes based on ClinVar database.
**Table S7.** Prior evidence of identified candidate variants based on published GWASs.
**Table S8.** Epigenomic information for candidate variants based on Roadmap database.
**Table S9.** Indicated mechanistic pathway for identified candidate variants.
**Table S10.** Estimated probability score for conserved region of the candidate variants.
**Table S11.** CpG annotation of candidate variants.
**Table S12.** The genes identified to be associated with SIH based on SKAT analysis.
**Table S13.** Morphometry quantification of the severity of acute pancreatitis.
**Table S14.** Comparison of distributions for clinical outcomes between rs1326680184 carriers and non rs1326680184 carriers.
**Table S15.** Comparison of distributions for laboratory measures between rs1326680184 carriers and non rs1326680184 carriers.
**Table S16.** Designation of Fcgbp‐targeted small hairpin RNA.

## Data Availability

The sequencing data have been deposited in GSA accession number HRA005131 (https://ngdc.cncb.ac.cn/gsa-human/browse/HRA005131). The data and scripts used are saved in GitHub https://github.com/qytangcq/WES_project. Supplementary materials (methods, figures, tables, graphical abstract, slides, videos, Chinese translated version, and update materials) could be found in the online DOI or iMetaOmics http://www.imeta.science/imetaomics/.
