# Supplementary material for: Whole exome sequencing reveals an FCGBP variant associated with spontaneous intraabdominal hemorrhage in severe acute pancreatitis
Source: IMetaOmics. 2025 Jan 9;2(1):e52. doi: 10.1002/imo2.52 (PMC12806392; doi:10.1002/imo2.52)
Supplement: Supplementary file 1 — Figure S1. Quantile‐quantile plot for the GWAS analysis of WES and SIH. Figure S2. Manhattan plot of genome‐wide association analysis for exonic variants in all autosomal chromosomes. Figure S3. Assay of serum FCGBP in patients with or without SIH. Figure S4. Immunohistochemical analysis of Fcgbp expression in lung tissue in mice. Figure S5. Immunohistochemical analysis of Fcgbp expression in vessel tissue in mice. Figure S6. Quantitative PCR analysis of FCGBP mRNA level in knockdown fibroblasts and wild‐type control. Figure S7. Relative expression levels of top down‐regulated genes and ECM‐related genes from RNA‐seq, assayed by qPCR. Figure S8. Single‐cell RNA‐sequencing analysis of fibroblasts. Figure S9. Pathology examination of lung and pancreas tissue of mice. Figure S10. Assays of blood MCP1, TNFα, and IL‐6 from mice induced as AP and normal healthy mice. [file IMO2-2-e52-s001.docx]

**Supporting information to**: **Whole Exome Sequencing Reveals an FCGBP Variant Associated with Spontaneous Intraabdominal Hemorrhage in Severe Acute Pancreatitis**

**Running title:** FCGBP and spontaneous intraabdominal hemorrhage in severe acute pancreatitis

Qiu-Yi Tang^1,2,3,#^, Yue-Peng Hu^2,4,#^, Qi Yang^2^, Jing Zhou^1,2^, Jing-Zhu Zhang^2^, Jie Yang^2^, Haibin Hao^2^, Gang Li^2^, Bai-Qiang Li^2^, Lu Ke^2^, Zhi-Hui Tong^1,2^, Yu-Xiu Liu^2^, Evan Yi-Wen Yu^5,6,*^, Wei-Qin Li^1,2,*^

^1^ School of Medicine, Southeast University, Nanjing 210009, China

^2^ Department of Critical Care Medicine, Jinling Hospital, Affiliated Hospital of Medical School, Nanjing University, Nanjing 210002, China

^3^ Department of General Surgery, Xinhua Hospital Affiliated to Shanghai Jiao Tong University School of Medicine, Shanghai 200092, China

^4^ Department of Emergency Medicine, The First Affiliated Hospital of Zhengzhou University, Zhengzhou, 450052, China

^5^ Key Laboratory of Environmental Medicine and Engineering of Ministry of Education, and Department of Epidemiology & Biostatistics, School of Public Health, Southeast University, Nanjing 210009, China

^6^ Department of Epidemiology, CAPHRI Care and Public Health Research Institute, School of Nutrition and Translational Research in Metabolism, Maastricht University, Maastricht 6229ER, the Netherlands

**^#^These authors contributed equally:** Qiu-Yi Tang, Yue-Peng Hu

**^*^Correspondence:** [liweiqindr@nju.edu.cn](file:///D:\Users\evanyu\Library\Containers\com.apple.mail\Data\Library\Mail%20Downloads\8292B98E-5401-4E03-8FD3-EC92B11F4A87\liweiqindr@nju.edu.cn) (Wei-Qin Li), [evan.yu@maastrichtuniversity.nl](mailto:evan.yu@maastrichtuniversity.nl) (Evan Yi-Wen Yu)

**Supplementary methods**

***Quality control of DNA sample in whole exome sequencing***

The genomic DNA extracted from peripheral blood was undertaken quality controls by: i) analysis of the degree of degradation and pollution of RNA with agarose gel electrophoresis; ii) test of the DNA purity with Nanodrop (Thermo Fisher Scientific, Waltham, MA), which is a spectrophotometer that enables highly accurate analyses; iii) meticulous quantification of DNA concentration with Qubit 3.0 (Thermo Fisher Scientific, Waltham, MA).

***Sequencing data processing including variants calling and quality control***

Sample reads mapping, variant calling, annotation, and quality control were performed using a pre-planned protocol, which were conducted in all participants irrespective of the outcomes (i.e., cases or controls). Quality control for raw FASTQ data was performed using software FastQc (version 0.11.9) to summarize statistics such as sequence count, sequence quality, GC content, adapter content, base N content, sequence length distribution, sequence duplication levels, and overrepresented sequence. MultiQC (version 1.14) was used to aggregate per-sample results into a single report. The sequence matched to the sequencing adapters and low-quality ends of reads (the Phred-scale quality score of base < 20) were removed with Trim-galore (version 0.6.7). The trimmed sequences were then interatly aligned to the human genome reference version hg38 (http://genome.ucsc.edu/) using Burrows-Wheeler Aligner (BWA) software (version 0.7.8-r455) with Maximum Entropy Method (BWA-MEM) algorithm; the output SAM files were transformed to binary format and indexed with SAMtools (version 1.9). Base score recalibration, local realignment for indel (insertion or deletion) detection, and duplication mark were performed with Genome Analysis Tool Kit (GATK, version 4.2.2.0, Broad Institute). The joint variant calling of germline variants was performed following GATK workflow for case group and control group, respectively, in which per-sample intermediate data were gathered and then produced a set of joint-called variants to enhance the sensitive detection of variants.

To validate the accuracy of variant calling obtained above, we additionally used a deep learning-based variant calling method, i.e., DeepVariant (version 1.3.0), to accomplish an independent procedure of variant calling, in which the two workflows (i.e., GATK and DeepVariant) performed largely equal on mapping the exome-wide variants.

The variants, with either missing rate > 0.50, minimal frequency of alternative alleles in case group < 3, Phred-scaled quality value < 30, or disqualified variant quality score recalibration (VQSR) (i.e., a new variant quality score named VQSLOD provides a continuous estimate of the probability that each variant is true and a threshold of 99.00 was applied), were removed using VCFtools (version 0.1.16).

***Functional annotation***

An in-silico approach through SNPnexus (<https://www.snp-nexus.org/v4/>), ANNOVAR (http://www.openbioinformatics.org/annovar), and RegulomeDB (https://beta.regulomedb.org/regulome-search/) was used to annotate the exonic variants. The allele frequencies of a given variant in large populations (particularly East Asians) were aligned with the Exome Aggregation Consortium database and 1000 Genomes Project database. Variants were defined as “uncommon” if they were present in < 1% of population in the reference databases. However, it is important to note that some variants, such as rs1326680184, did not have available allele frequency information in the genome reference database and were consequently not filtered out from further analysis. Genetic variants predicted to be deleterious were identified if they disrupt the protein-coding sequence (refer to nonsynonymous, stop-gained, start-loss, frameshift, or canonical splicing-site alteration). In addition, to examine predicted functional impact, particularly for the frameshit mutations that could not be annotated by neither Polymorphism Phenotyping (PolyPhen) nor Sorting Intolerant from Tolerant algorithm (SIFT) designed for single-nucleotide polymorphisms (SNPs), a combined annotation dependent depletion (CADD) method was annotated to the variants (Phred scores > 20 predicted as deleterious, <https://cadd.gs.washington.edu/score>).

***ELISA***

Concentrations of human serum FCGBP were detected with a Human FCGBP ELISA Kit (RK01369, ABclonal, China) following the manufacturer’s instructions, and compared between FCGBP variant carriers and non-carriers by Student’s t test with Welch’s correction. Mouse serum levels of MCP1, IL-6 and TNFα were measured using MCP1, IL-6 and TNFα ELISA kits (RK00381, RK00008, RK00027, ABclonal, China), which the differences between AP mice and control mice were compared using Man-Whitney U test.

***Western-blot***

Briefly, tissues from mice were lysed in RIPA lysis buffer, and total proteins were extracted. Concentrations of proteins were measured using the BCA protein assay kit (Pierce, Thermo Fisher Scientific) following the commercial instructions. Equivalent protein (approximately 30 μg/sample) was separated by SDS-PAGE gel and then transferred to PVDF membranes. Then the membranes were blocked for 1 hour in 5% nonfat milk. Afterwards, membranes were incubated with primary antibodies against GAPDH (1:1,000 dilution; Sigma-Aldrich), Fcgbp (1:1,000 dilution; Bioss) overnight at 4°C. Finally, HRP-conjugated secondary antibodies were applied for 1 hour. The proteins were detected and analyzed by an ECL Plus chemiluminescence imaging system (Tanon). The quantified results of Western-blot were compared between Fcgbp-KD mice and control mice using Man-Whitney U test.

Please see **Table S1-S16** in the additional Excel file.

**
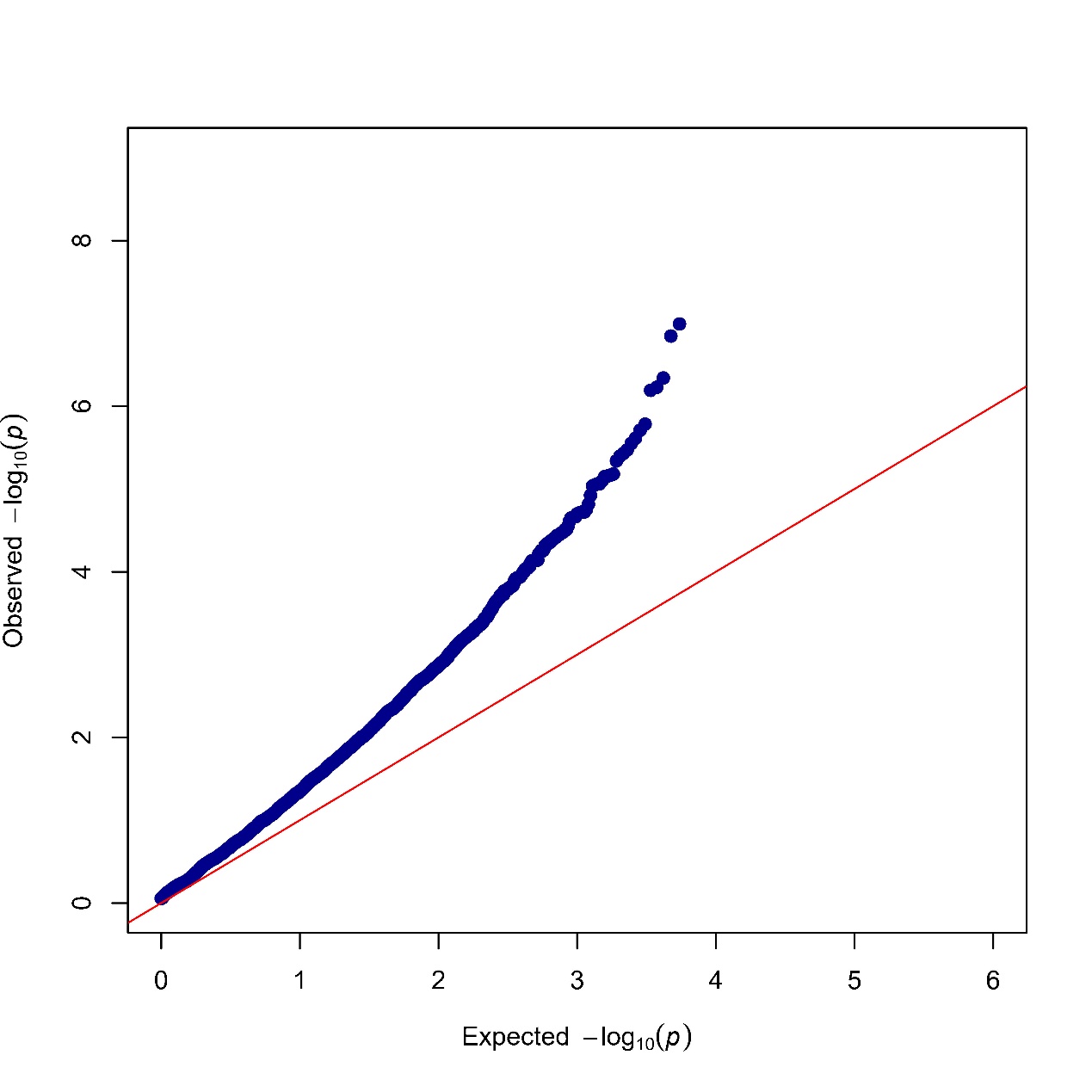
**

**Figure S1 Quantile-quantile plot for the GWAS analysis of WES and SIH.**

The y axis indicates the actual -log_10_ (*p* value of each variant) and the x axis indicates the expected -log_10_ (*p* value of each variant).

Abbreviations: GWAS, genome-wide association study; WES, whole exome sequence; SIH, spontaneous intraabdominal hemorrhage.

**
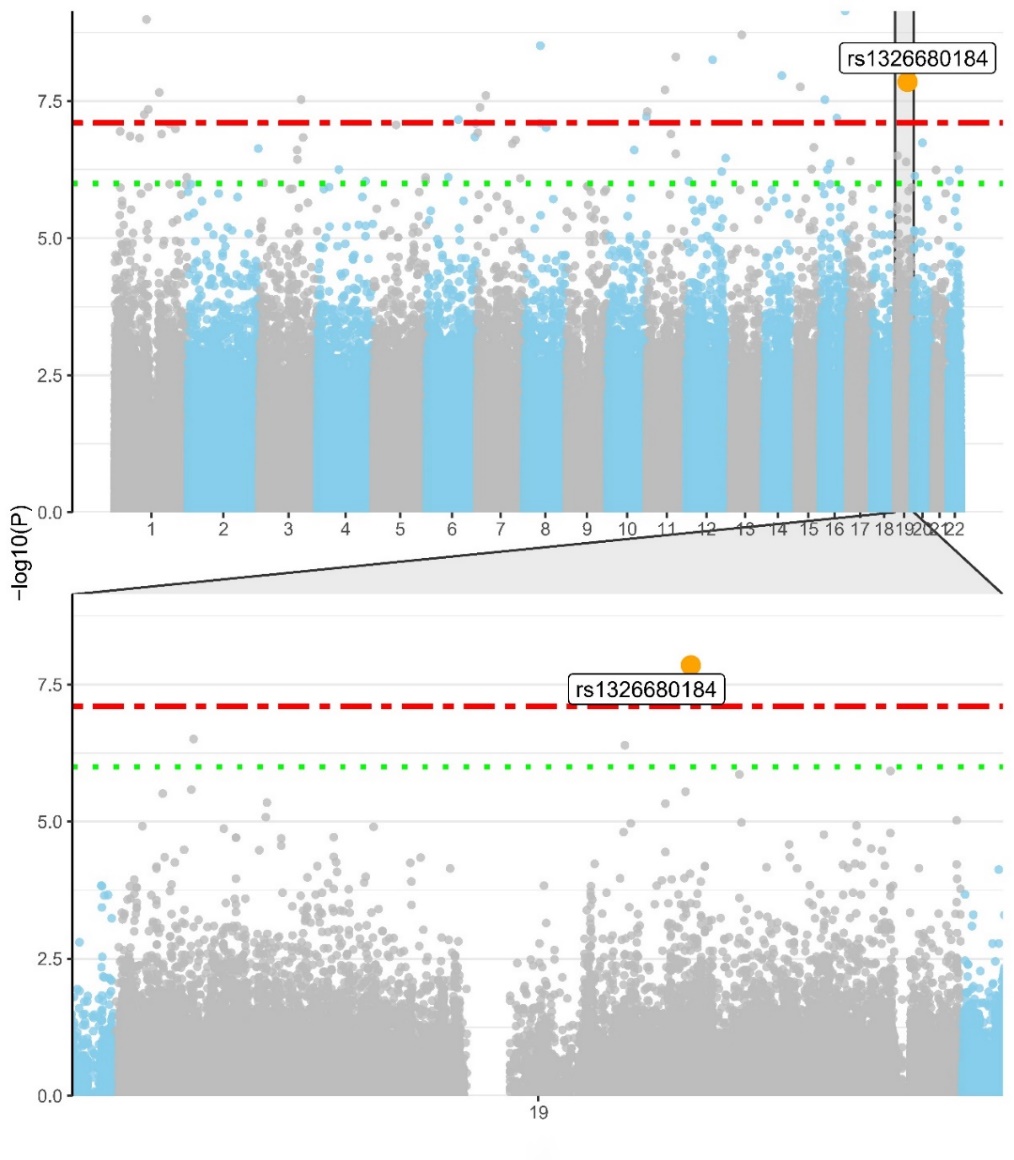
**

**Figure S2 Manhattan plot of genome-wide association analysis for exonic variants in all autosomal chromosomes.**

The genome-wide significance was set at 5×10^-6^ (5×10^-8^/1×10^-2^ as exome accounts for approximately 1% of human genome).

The rs1326680184 was used and denoted to represent the identified six *FCGBP* variants.


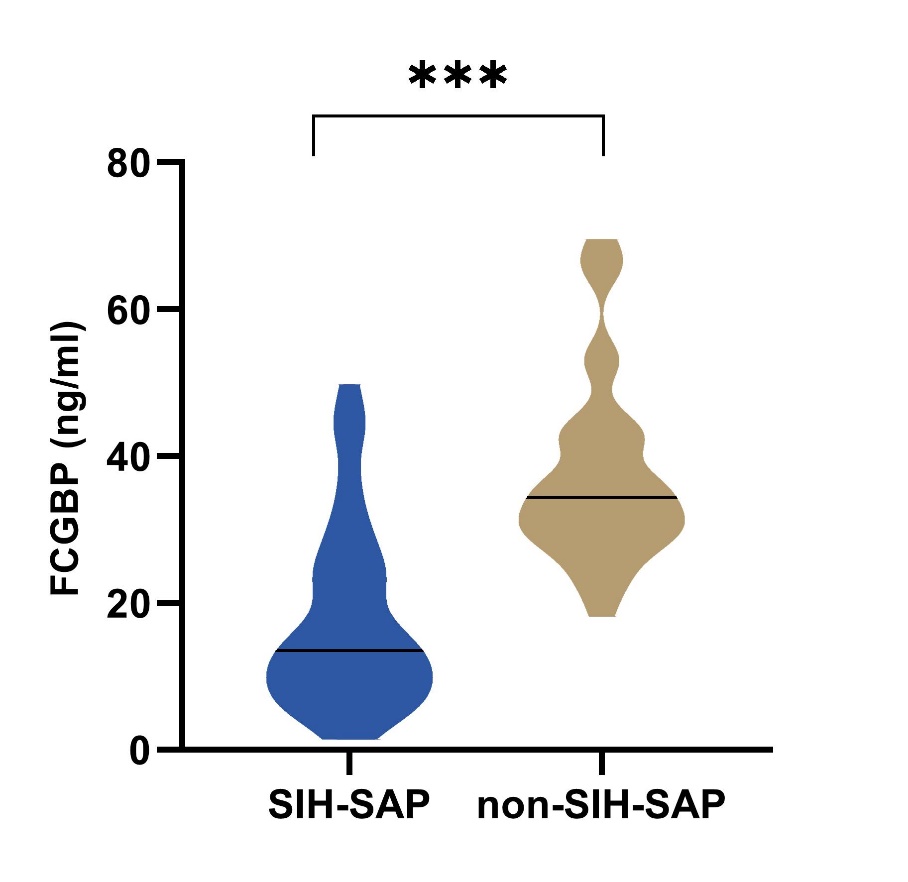


**Figure S3 Assay of serum FCGBP in patients with or without SIH**

*** indicates p < 0.001 by Mann-Whitney test.


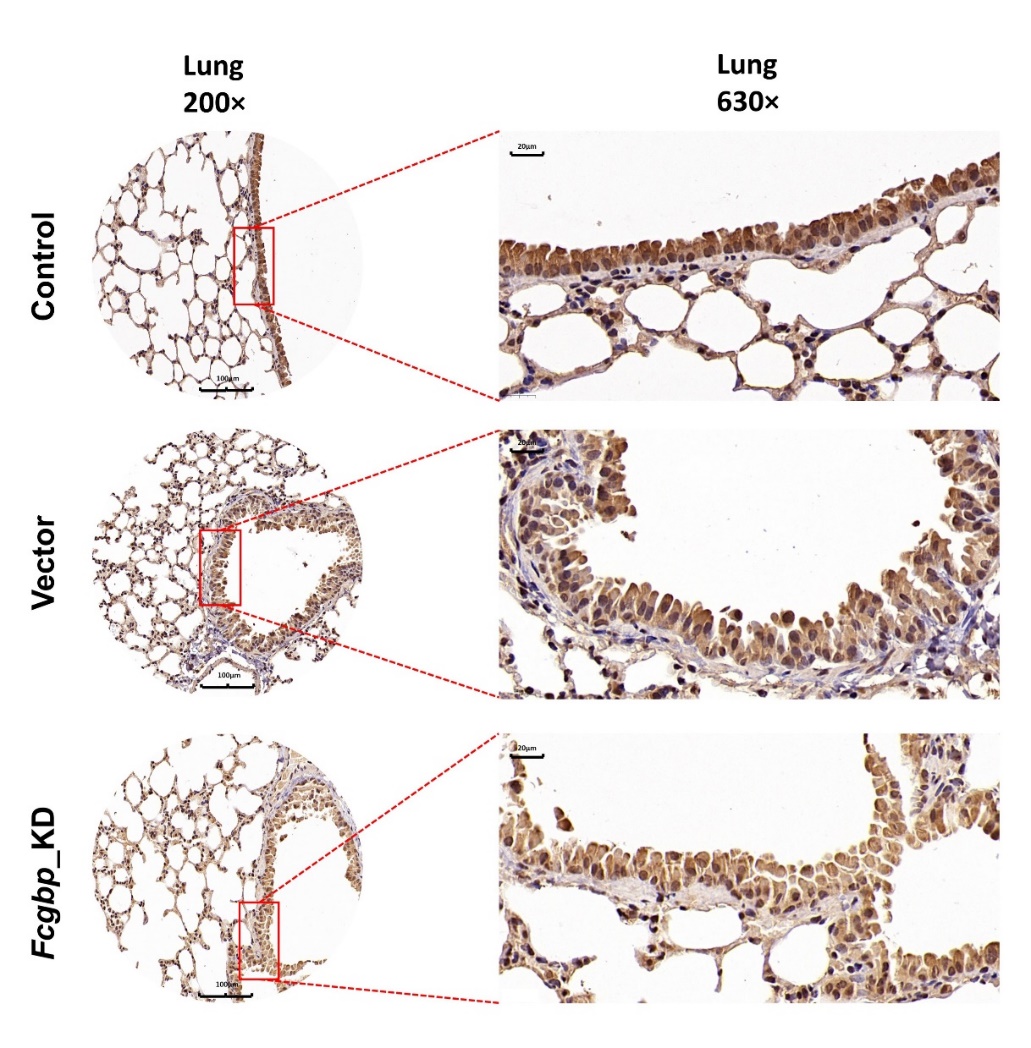


**Figure S4 Immunohistochemical analysis of Fcgbp expression in lung tissue in mice.**

Representative IHC images of Fcgbp in lung tissue were shown (n = 10 in each group), which indicates the injection of AAV-RNAi successfully reduces the expression of *Fcgbp* levels in lung tissue in mice.

Abbreviations: IHC, immunohistochemistry; AAV-RNAi, adeno-associated virus harboring short.

**
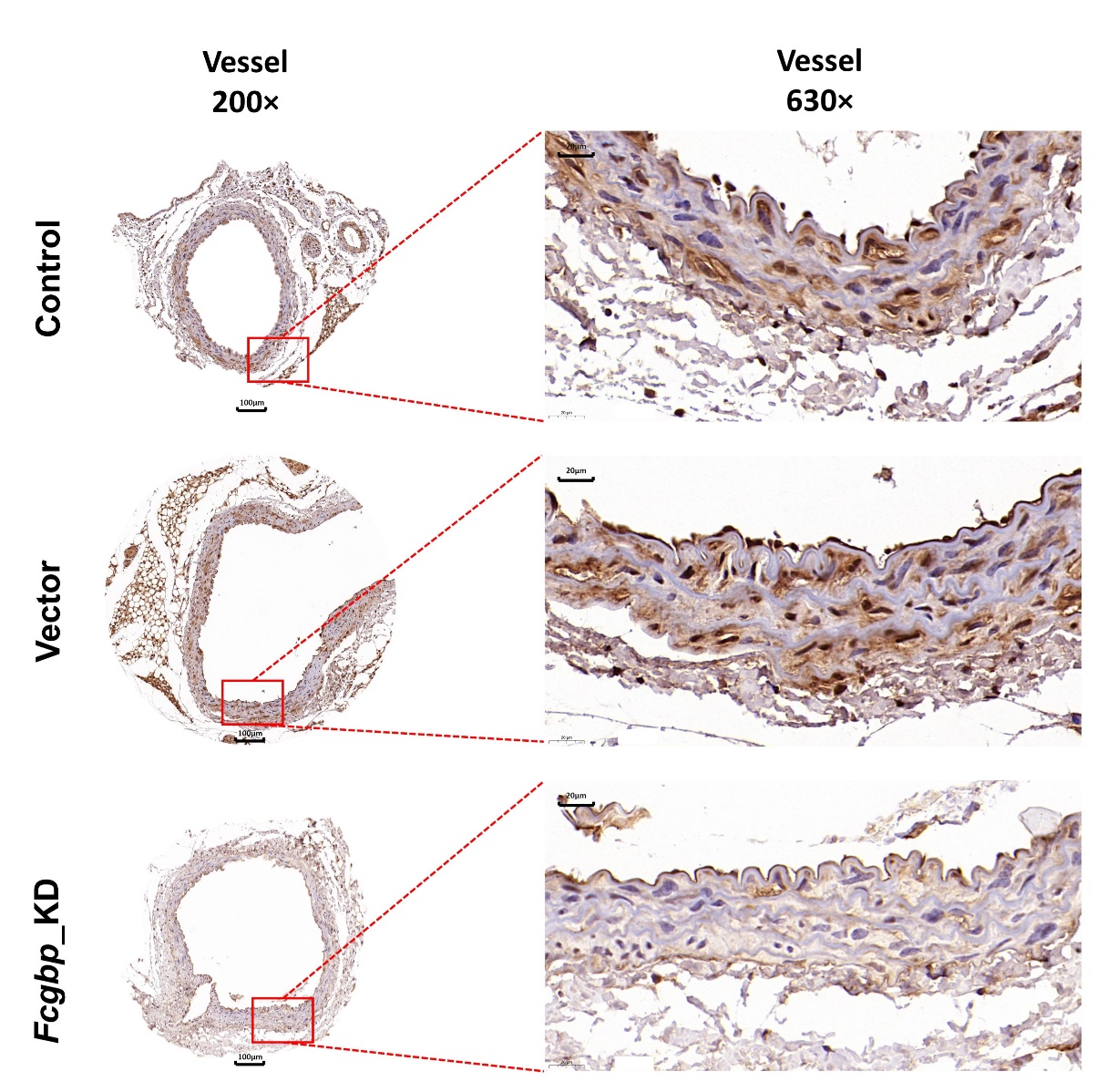
**

**Figure S5 Immunohistochemical analysis of Fcgbp expression in vessel tissue in mice.**

Representative IHC images of Fcgbp in vessel tissue were shown (n = 10 in each group), which indicates the injection of AAV-RNAi successfully reduces the expression of *Fcgbp* levels in tissue of vessel in mice.

Abbreviations: IHC, immunohistochemistry; AAV-RNAi, adeno-associated virus harboring short.

**
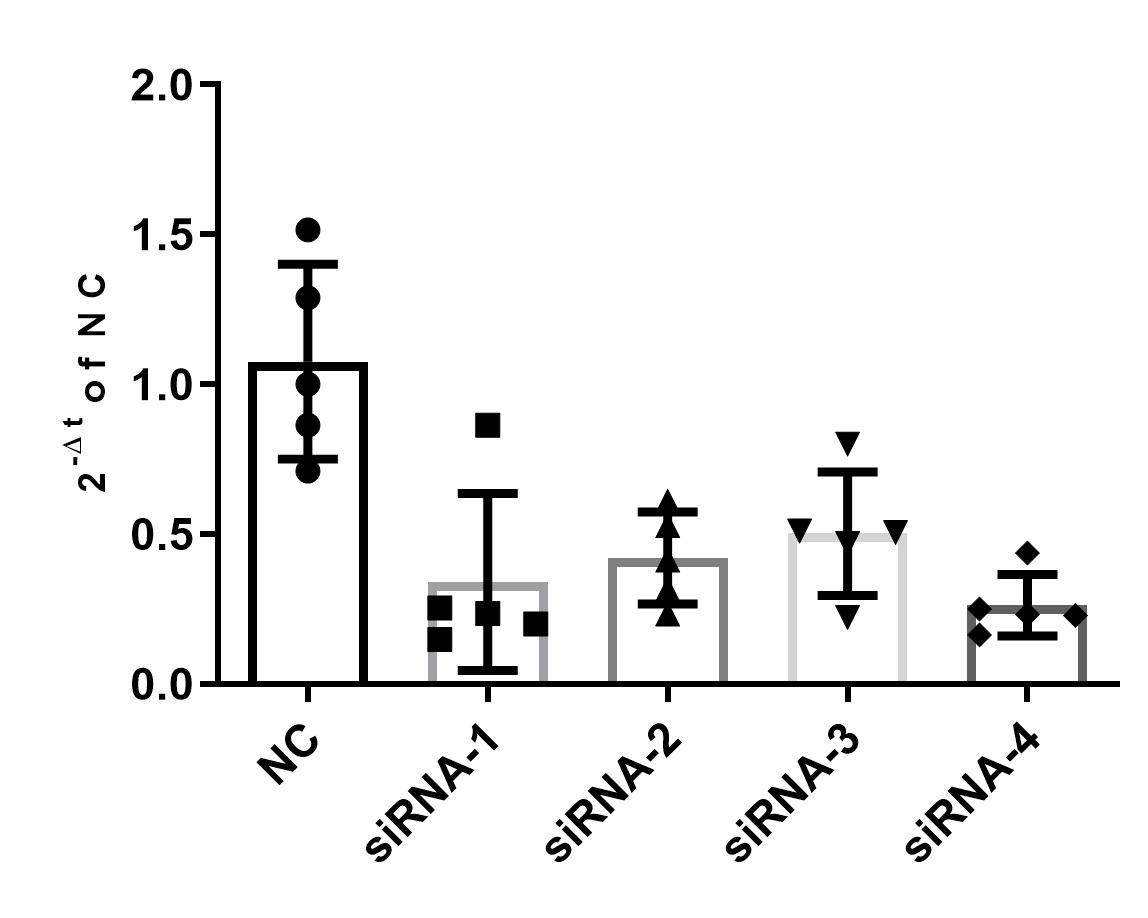
**

**Figure S6 Quantitative PCR analysis of *FCGBP* mRNA level in knockdown fibroblasts and wild-type control.**

Abbreviations: PCR, polymerase chain reaction.

**
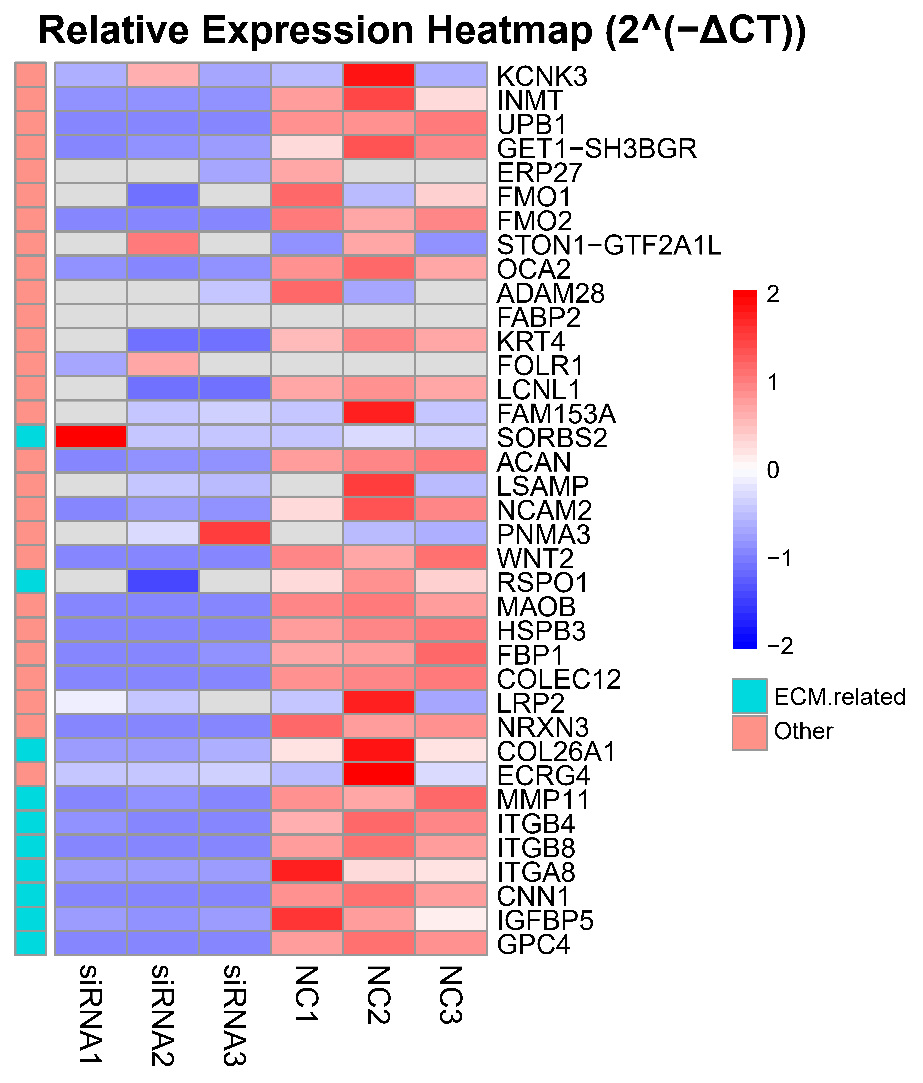
Figure S7 Relative expression levels of top down-regulated genes and ECM-related genes from RNA-seq, assayed by qPCR**

Abbreviations: ECM, extracellular matrix; PCR, polymerase chain reaction.

**
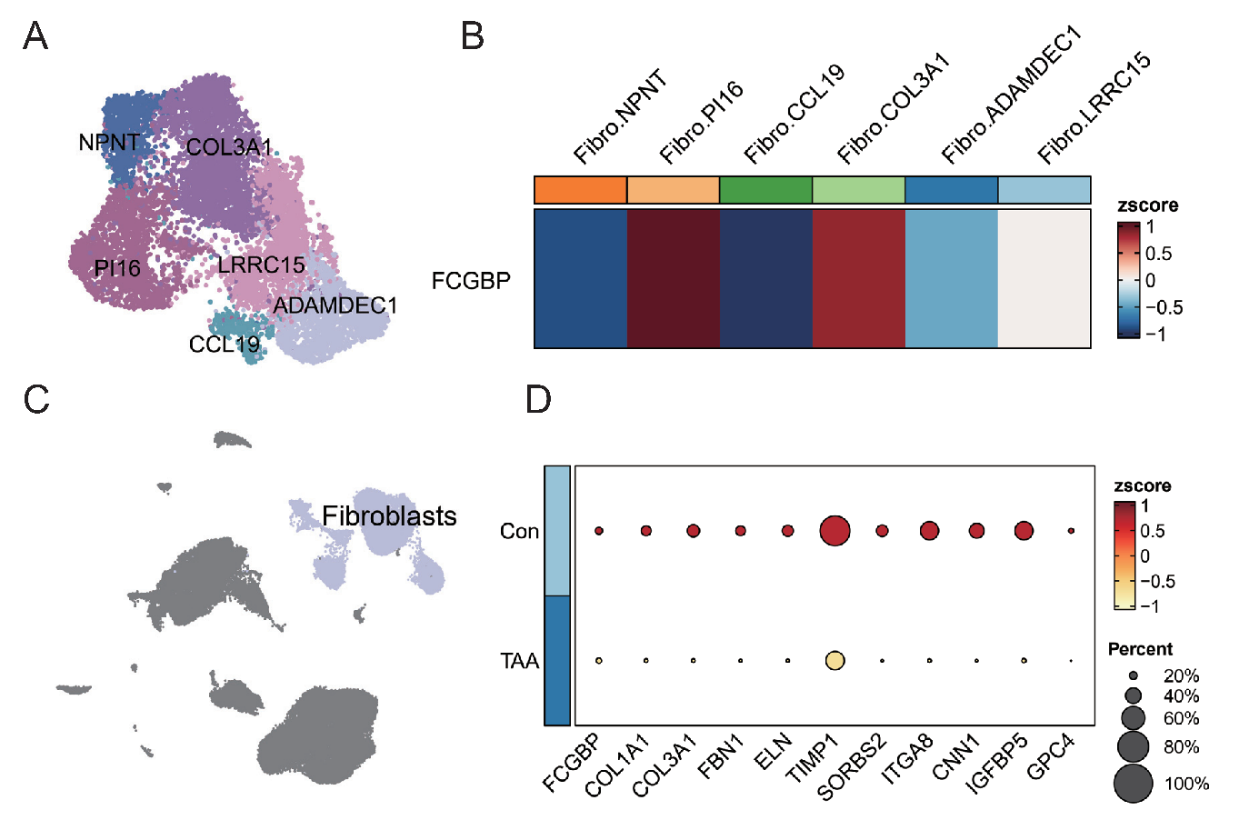
Figure S8** **Single-cell RNA-sequencing analysis of fibroblasts.**

(A) Dimension reduction plot of fibroblast clusters from pancreatic cancer samples; (B) Heatmap of *FCGBP* expression across subtypes of fibroblasts; (C) Dimension reduction plot of cells from thoracic aorta aneurysm samples and normal aorta samples, fibroblasts were highlighted; (D) Dot plot illustrating the expression of extracellular matrix-related genes and *FCGBP* between thoracic aorta aneurysm samples and control samples.

**
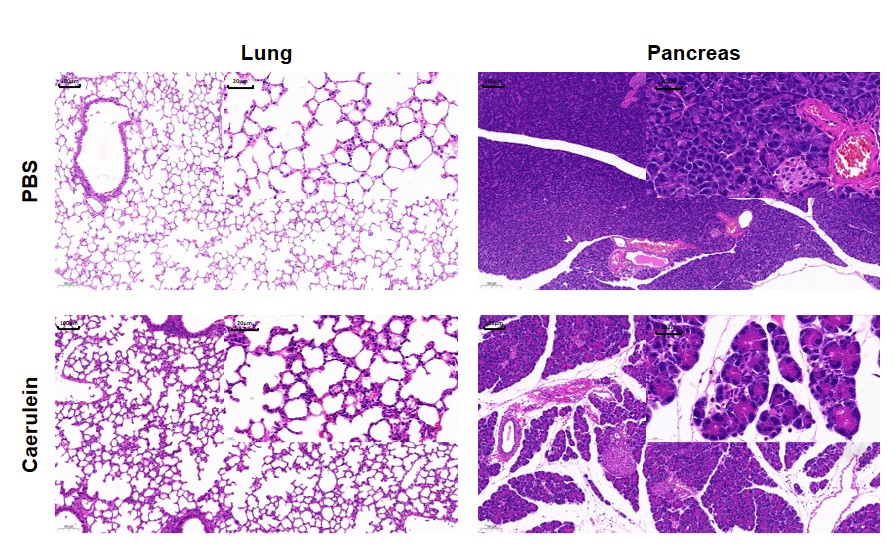
 Figure S9 Pathology examination of lung and pancreas tissue of mice.**

The pathological examination of mice induced as acute pancreatitis presented acinar cell necrosis, endo-cellular and extra-cellular edema, infiltration of neutrophils in pancreas tissue, and severe damage of alveoli. Abbreviations: PBS, Phosphate Buffer Saline.

**
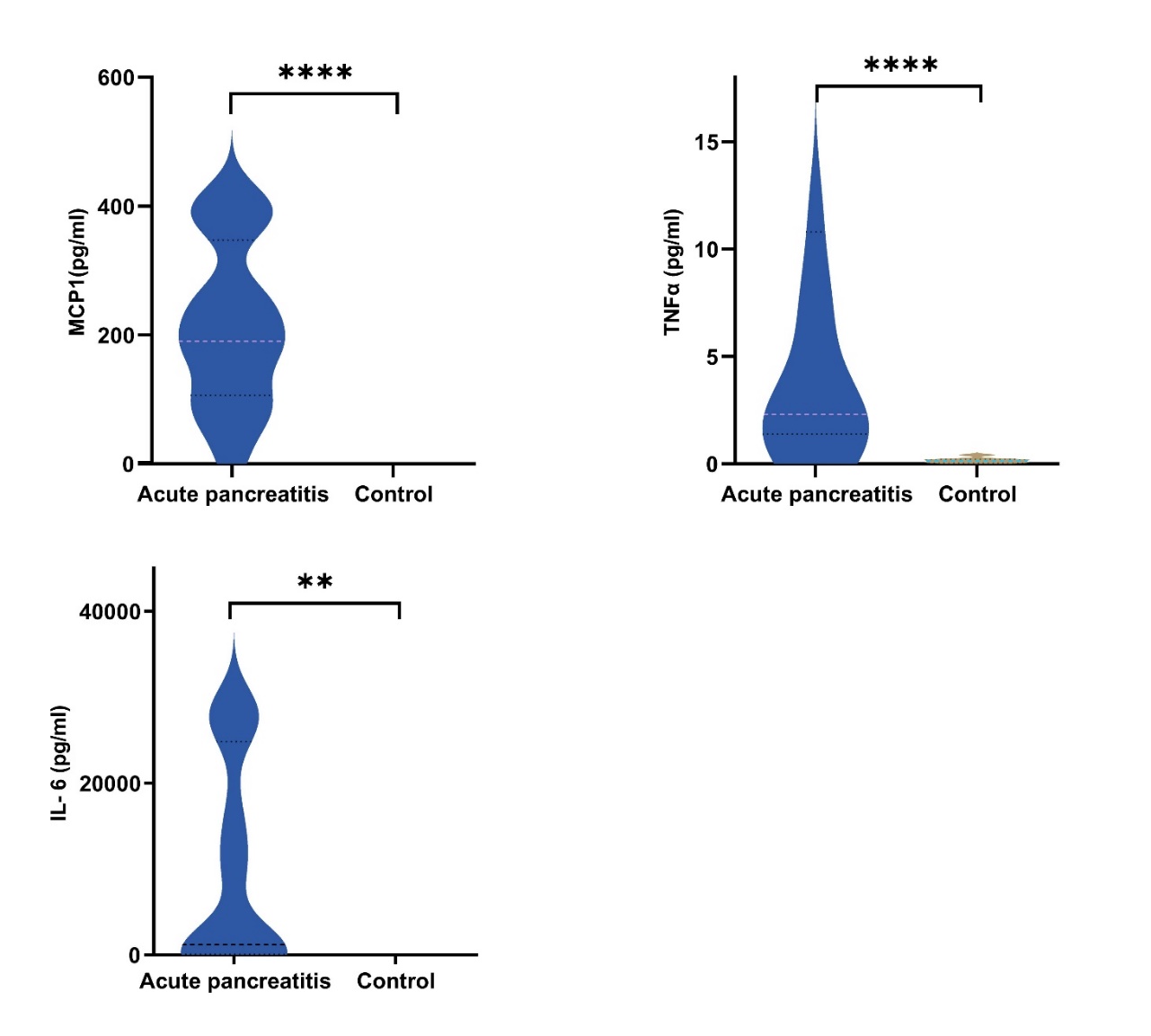
**

**Figure S10 Assays of blood MCP1, TNFα and IL-6 from mice induced as AP and normal healthy mice.**

** indicates p < 0.01, **** indicates p < 0.0001 by Mann-Whitney test
